# Species-specific inflammatory responses of chondrocytes: Toward evidence-based model selection in OA research

**DOI:** 10.1016/j.isci.2026.117710

**Published:** 2026-09-22

**Authors:** Angkana Kidtiwong, Maria-Belen Arteaga, Karyna Tarasova, Sinan Gültekin, Mario Rothbauer, Iris Gerner, Florien Jenner

**Affiliations:** 1 Department for Companion Animals and Horses, University Equine Hospital, University of veterinary medicine Vienna, Vienna, Austria; 2 Department of Orthopedics and Trauma-Surgery (Division of Orthopedics), Medical University, Vienna, Vienna, Austria; 3 Austrian Cluster for Tissue Regeneration, Vienna, Austria

**Keywords:** 3R, animal model, chondrocyte, inflammation, osteoarthritis, translational gap

## Abstract

To inform evidence-based osteoarthritis model selection, this study compares inflammatory responses of articular chondrocytes from six species (human, horse, sheep, rabbit, rat, and mouse) under standardized IL-1β and TNF-α stimulation in 2D and 3D culture. Chondrocytes were assessed for viability, proliferation, wound healing, and transcriptomic changes via RT-qPCR and 3′RNA sequencing. Marked interspecies differences were observed in morphology, proliferation, and inflammatory gene activation, with only two genes (*SOD2*, *NFKBIA*) conserved across all species. Human, horse, and rat chondrocytes shared pathways including acute phase response, deubiquitination, and class-A/1-GPCR signaling. Species-specific responses included matrix remodeling in horses, rheumatoid arthritis-related pathways in sheep, and mixed inflammatory-regenerative profiles in rats. While no species fully replicates human OA, equine and rat chondrocytes best reflect key aspects of human inflammation. These findings underscore the need for molecular criteria in model selection and advocate for human cell-based models when interspecies differences limit the translational value of animal models.

## Highlights

• Species-specific chondrocyte responses challenge translational assumptions
• No single animal model fully replicates human osteoarthritis inflammation
• Species share only 2 (SOD2, NFKBIA) of 463 differentially expressed genes
• Endotype fidelity—not phenotype—should guide model selection

## Graphical abstract

**Figure:**
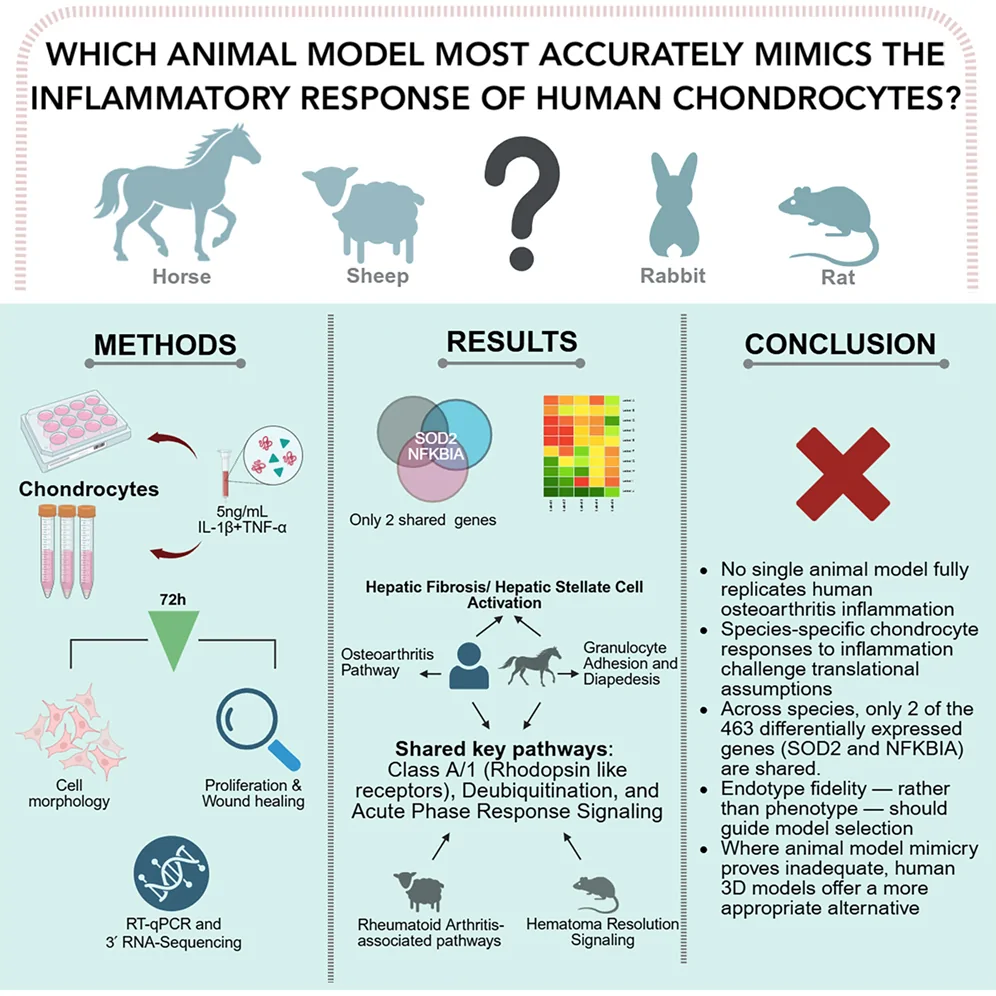

## Teaser

Immunology; Transcriptomics

## Introduction

Osteoarthritis (OA) is a heterogeneous disease encompassing diverse clinical phenotypes and underlying endotypes, yet inflammation is a key driver of OA pathogenesis across all molecular subtypes.^1^^,^^2^^,^^3^ Pro-inflammatory cytokines are consistently upregulated in osteoarthritic joints, and contribute to cartilage degeneration, subchondral bone remodeling, and synovial inflammation by orchestrating signaling cascades that promote catabolic activity, inhibit tissue repair, and alter chondrocyte phenotype and function.^1^^,^^2^^,^^3^ Among these cytokines, interleukin-1β (IL-1β) and tumor necrosis factor-α (TNF-α) are central mediators of OA-associated inflammation, activating NF-κB and inducing the expression of matrix-degrading enzymes (e.g., *MMP*s), inflammatory mediators (e.g., IL-6) and nociceptive signaling pathways.^1^^,^^2^^,^^3^^,^^4^^,^^5^ Simultaneously, IL-1β and TNF-α suppress the synthesis of key extracellular matrix (ECM) components, including aggrecan and type 2 collagen, thereby disrupting cartilage homeostasis and accelerating structural deterioration.^1^^,^^2^^,^^3^^,^^4^^,^^5^ Stimulation with IL-1β and TNF-α is therefore widely employed to model OA-associated inflammation both *in vitro* and *in vivo*, as it induces gene expression changes in human chondrocytes that closely resemble those observed in OA.^4^^,^^5^

Despite extensive research efforts, no disease-modifying osteoarthritis drugs (DMOADs) have been successfully translated into clinical practice. A major barrier to therapeutic progress is the limited predictive value of preclinical animal models, which continues to impede the translation of preclinical findings to human disease.^5^^,^^6^ Indeed, more than 90% of OA therapies showing promise in preclinical studies ultimately fail in clinical trials, most commonly due to lack of efficacy (52%) or safety concerns (24%).^6^^,^^7^^,^^8^^,^^9^^,^^10^^,^^11^^,^^12^ This high attrition rate, coupled with poor reproducibility, affecting 51%–89% of preclinical studies, undermines scientific advances and leads to significant ethical and economic consequences.^6^^,^^7^^,^^8^^,^^9^^,^^10^^,^^11^^,^^12^

Yet the reliance on animal models to investigate molecular mechanisms, evaluate therapeutic interventions, and assess safety profiles prior to human trials persists.^5^^,^^6^^,^^13^^,^^14^ This dependence is further reinforced by the limited availability of human joint tissue, which is often collected during arthroplasty and thus reflects end-stage OA, precluding insights into early disease pathogenesis. However, the prevailing approach to model selection has traditionally prioritized logistical and technical considerations—such as cost, animal size, ease of handling, speed of disease induction, and amenability to genetic modification—over mechanistic fidelity to human disease.^10^^,^^11^^,^^12^^,^^14^^,^^15^ Consequently, many existing animal models primarily phenocopy OA, exhibiting similar clinical symptoms and structural outcomes (e.g., cartilage degradation, osteophyte formation), but fail to accurately mimic the complex molecular and cellular processes that drive naturally occurring disease pathogenesis and progression.^10^^,^^11^^,^^12^^,^^14^^,^^15^ Furthermore, even the macroscopic and microscopic manifestations of OA differ sufficiently between species that species-specific scoring systems have been developed.^16^^,^^17^^,^^18^^,^^19^^,^^20^^,^^21^^,^^22^^,^^23^^,^^24^^,^^25^ Such variation in morphological presentation strongly suggests corresponding divergence in the underlying molecular and cellular mechanisms, underscoring the need for systematic molecular endotyping across species. Lacking alignment of underlying pathomechanisms between models and human disease due to inherent interspecies differences in chondrocyte inflammatory responses, can impede the identification of effective therapeutic targets and biomarkers for human OA and lead to misleading preclinical efficacy. Such interspecies variations in inflammatory signaling are well-documented in other tissues, affecting fundamental processes such as Toll-like receptor (TLR) signaling cascades, the precise repertoire and kinetics of cytokine production (e.g., IFN-γ, IL-10), and the responsiveness of immune cell populations to pro-inflammatory stimuli, which can significantly affect drug metabolism and receptor affinities.^26^^,^^27^^,^^28^^,^^29^^,^^30^^,^^31^^,^^32^ Crucially, species-specific differences in chondrocytes’ inflammatory responses at a molecular level have not yet been systematically examined across a broad range of commonly used animal models in direct comparison to humans.

Therefore, bridging the translational gap demands that each animal model’s inflammatory response be rigorously validated against the human baseline, aligning preclinical approaches with human OA biology and pathways underlying disease pathogenesis. Building upon established evidence of species-specific inflammatory pathway activation and response patterns observed in other tissues, we hypothesize that the IL-1β and TNF-α-driven transcriptional activation of key inflammatory and catabolic pathways in chondrocytes will exhibit distinct species-specific patterns, impacting their suitability as translational models for human OA. To address this, our study systematically compares chondrocyte responses to standardized inflammatory stimuli (IL-1β and TNF-α) across commonly used OA animal model species—namely mice, rats, rabbits, sheep, and horses^5^^,^^14^^,^^33^^,^^34^^,^^35^^,^^36^^,^^37^^,^^38^^,^^39^—in both two-dimensional (2D) and three-dimensional (3D) culture systems. Our aim is to identify the most suitable models to enhance therapeutic development and clinical translation for both human and veterinary applications, and to support the 3R principles (replacement, reduction, and refinement) in animal research.

## Results

### Morphology

Phalloidin/DAPI staining revealed distinct interspecies differences in chondrocyte morphology. Overall cell size, nucleus size, cytoplasm size, nucleus/cytoplasm ratio, and cell circularity varied significantly between species (*p* < 0.0001). Equine chondrocytes were notably smaller and more compact, while murine cells were largest (Figure 1B). Human cells were significantly smaller than murine chondrocytes (*p* < 0.0001) but larger than equine cells (*p* = 0.0164) and had smaller nuclei and cytoplasm than mice (*p* = 0.0012, *p* < 0.0001) but larger than sheep (*p* = 0.0054, *p* = 0.041) and horses (*p* = 0.003, *p* = 0.0033). Human chondrocytes also displayed a lower nucleus-to-cytoplasm ratio than horse (*p* < 0.0001) and sheep (*p* = 0.0002) and lower circularity than rat (*p* = 0.008, Figures 1C and 1D).

**Figure 1:**
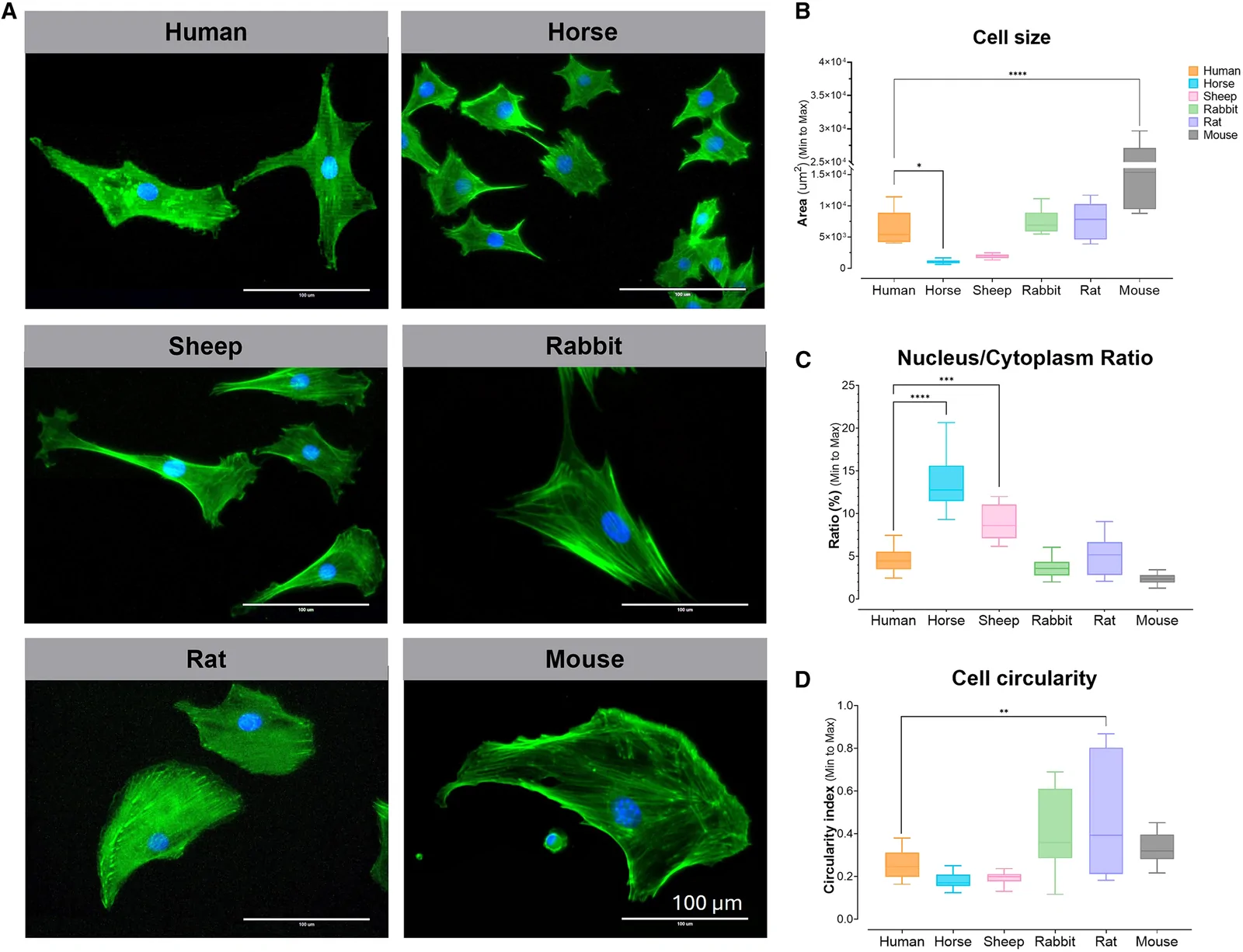
Morphological comparison of chondrocytes across species Representative images of phalloidin and DAPI-stained chondrocytes from five animal species and human, imaged at 40× magnification across 10 fields of view. Scale bars, 100 μm, are shown and apply to all representative micrographs. Boxplots (center line: median, box: interquartile range (25^th^–75^th^ percentile), whiskers: minimum to maximum values) show quantitative analysis of cell size, nucleus-to-cytoplasm ratio, and cell circularity. Statistical comparison to human cells was performed using ordinary one-way ANOVA. Horse chondrocytes were significantly smaller, whereas mouse chondrocytes were significantly larger than human (∗*p* < 0.05, ∗∗∗∗*p* < 0.0001). Human chondrocytes demonstrated significantly lower nucleus-to-cytoplasm ratio compared to horse (∗∗∗∗*p* < 0.0001) and sheep (∗∗∗*p* < 0.001). Rat chondrocytes displayed significantly greater circularity than human (∗∗*p* < 0.01).

### Murine chondrocyte apoptosis

Murine chondrocytes showed evidence of passaging-induced apoptosis, indicated by increased caspase 3/7 activity (Figure S1) and were therefore excluded from subsequent assays (details additional assay for mouse; SA-β galactocidase and caspase 3/7 activity: see supplemental information).

### Inflammation dose titration and cell viability

Quantitative reverse-transcription PCR (RT-qPCR) showed species-specific thresholds for *IL-6* and *MMP1* induction (Figures 2A and 2B; Table S1). While human and sheep chondrocytes significantly upregulated *MMP1* at 1 ng/mL IL-1β and TNF-α, rabbit and rat chondrocytes responded at ≥2.5 ng/mL, and horse cells only at ≥5 ng/mL. Similarly, *IL-6* expression was significantly increased in human, sheep, and rabbit chondrocytes at 2.5 ng/mL, whereas rat and horse cells required ≥5 ng/mL to elicit a comparable response.

**Figure 2:**
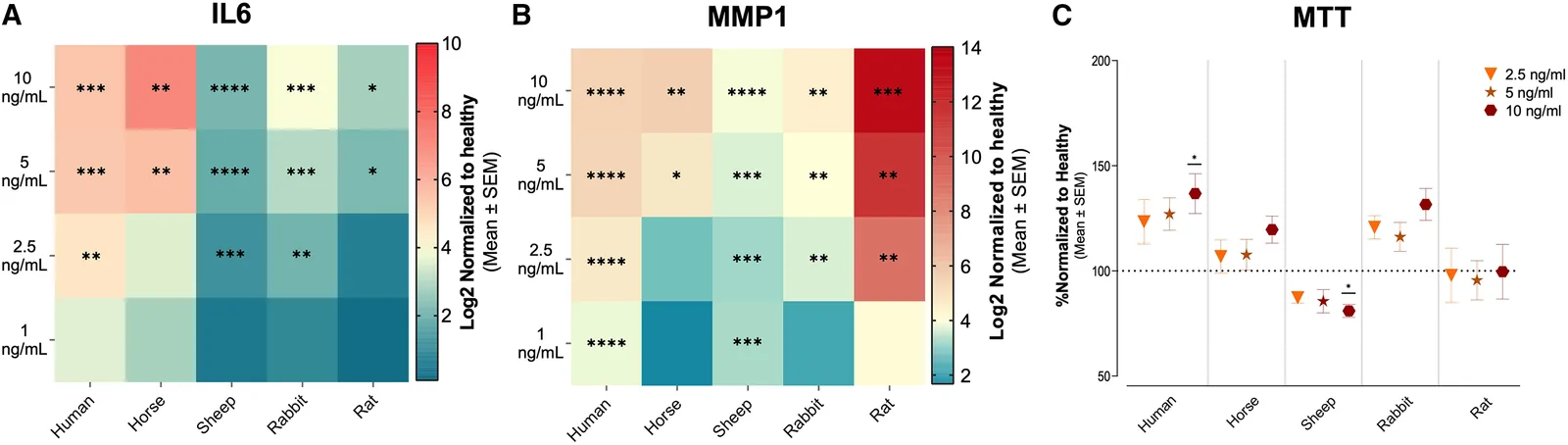
Dose titration of inflammatory concentrations across species This figure illustrates the dose-response of four different concentrations of inflammatory stimuli on chondrocytes in 2D cell culture system across five species (*n* = 3 donors). The data shown as mean ± SEM of log_2_ fold change of key inflammatory markers relative to healthy control (A and B). One-way ANOVA was used to assess a significance level. Following exposure to 5 and 10 ng/mL doses, *IL-6*, and *MMP1* expression levels were significantly upregulated in all species. Notably, while 10 ng/mL of inflammatory cytokines markedly stimulated metabolic activity in human chondrocytes, this same dose significantly reduced metabolic activity in sheep chondrocytes (C). (∗*p* < 0.05, ∗∗*p* < 0.01, ∗∗∗*p* < 0.001, and ∗∗∗∗*p* < 0.0001). See also Table S1.

Given that a dose of 10 ng/mL IL-1β and TNF-α reduced metabolic activity in sheep chondrocytes while enhancing it in human cells, as shown by 3-(4,5-dimethylthiazol-2-yl)-2,5-diphenyltetrazolium bromide (MTT) assays (Figure 2C), a concentration of 5 ng/mL was selected for standardized inflammatory stimulation in downstream experiments.

### Proliferation assay

Chondrocyte proliferation rates demonstrated significant interspecies differences (*p* = 0.0042), evident under both healthy (*p* = 0.0217) and inflamed (*p* = 0.0064) conditions. Overall, equine chondrocytes proliferated fastest, while rabbit and human cells showed the slowest growth (Figures 3A, 3C, and 3E). Specifically, under healthy conditions, rabbit chondrocytes proliferated significantly slower than horse (*p* = 0.006) and sheep (*p* = 0.0401). When inflamed, rabbit cells maintained significantly slower proliferation compared to horse (*p* = 0.0003) and sheep (*p* = 0.045). Additionally, inflamed horse chondrocytes proliferated significantly faster than human cells (*p* = 0.0012). Notably, inflammatory stimulation did not alter proliferation compared to healthy chondrocytes in most species, except for horses, where it led to a significant increase in cell growth (*p* = 0.0094; Figure 3E).

**Figure 3:**
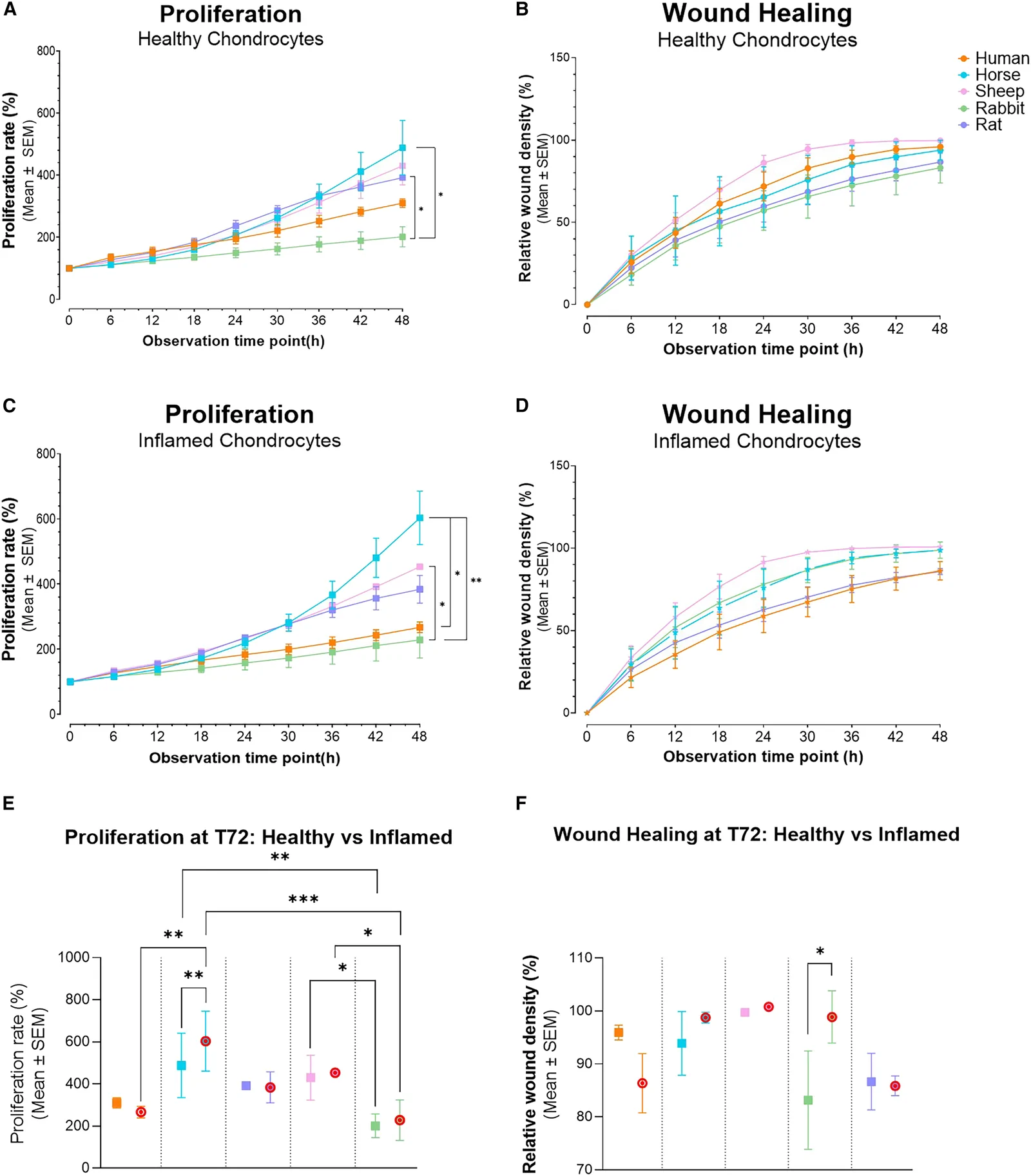
Time course of chondrocyte proliferation and wound healing across species: T24-T72 (A, C, and E) Proliferation rates and (B, D, and F) relative wound density were monitored over 48 h under healthy (A and B) and inflamed (C and D) conditions). Data are presented as mean ± SEM of the percentage of proliferation rate and relative wound density from three biological donors (*n* = 3). Comparison of the proliferation and wound healing rate of healthy and inflamed (indicated by red circles) chondrocytes at 48 h of observation (T72 time point) (E and F). Percent change in cell confluence relative to the initial time point (0 h) and relative wound density were recorded every 6 h. Statistical comparisons were performed using ANOVA. (∗*p* < 0.05, ∗∗*p* < 0.01). See also Figure S3.

### Wound healing scratch assay

Wound closure rate did not show any significant differences between species (*p* = 0.1682, Figure 3B) or between healthy and inflamed chondrocytes (*p* = 0.3592, Figure 3D), except in rabbit chondrocytes, which showed significantly faster wound closure when inflamed (*p* = 0.0135, Figure 3F).

### Comparative transcriptomic analysis of inflamed chondrocytes across species: NGS

RNA sequencing (RNA-seq) identified 463 differentially expressed genes (DEGs) in human (Table S2), 72 in horse (Table S3), 152 in sheep (Table S4), and 65 in rat (Table S5) chondrocyte pellets after 5 ng/mL stimulation. Rabbit chondrocyte pellets did not yield any significant DEGs following 5 ng/mL inflammatory stimulation and were therefore excluded from downstream analyses including the Venn diagram and ingenuity pathway analysis (IPA).

Only *SOD2* and *NFKBIA* were consistently differentially expressed across all four species, highlighting conserved inflammatory stress and NF-κB pathway involvement. Sheep shared the highest number of DEGs with human (*n* = 31 [of 152], 20.4%), followed by horse (*n* = 26 [of 72], 36.1%), and rat (*n* = 23 [of 65], 35.4%, Figure 4A). Six genes (*BIRC3*, *MMP1*, *MMP13*, *FTH1*, *PDGFRA*, and *AEBP1*) were upregulated in human, horse, and sheep; human-rat overlaps included *IL6*, *MMP3*, *MMP9*, *CXCL3*, *HMOX1*, *MT2A*, *COMP*, *PRG4*, *COL1A2*, and *CCN2*.

**Figure 4:**
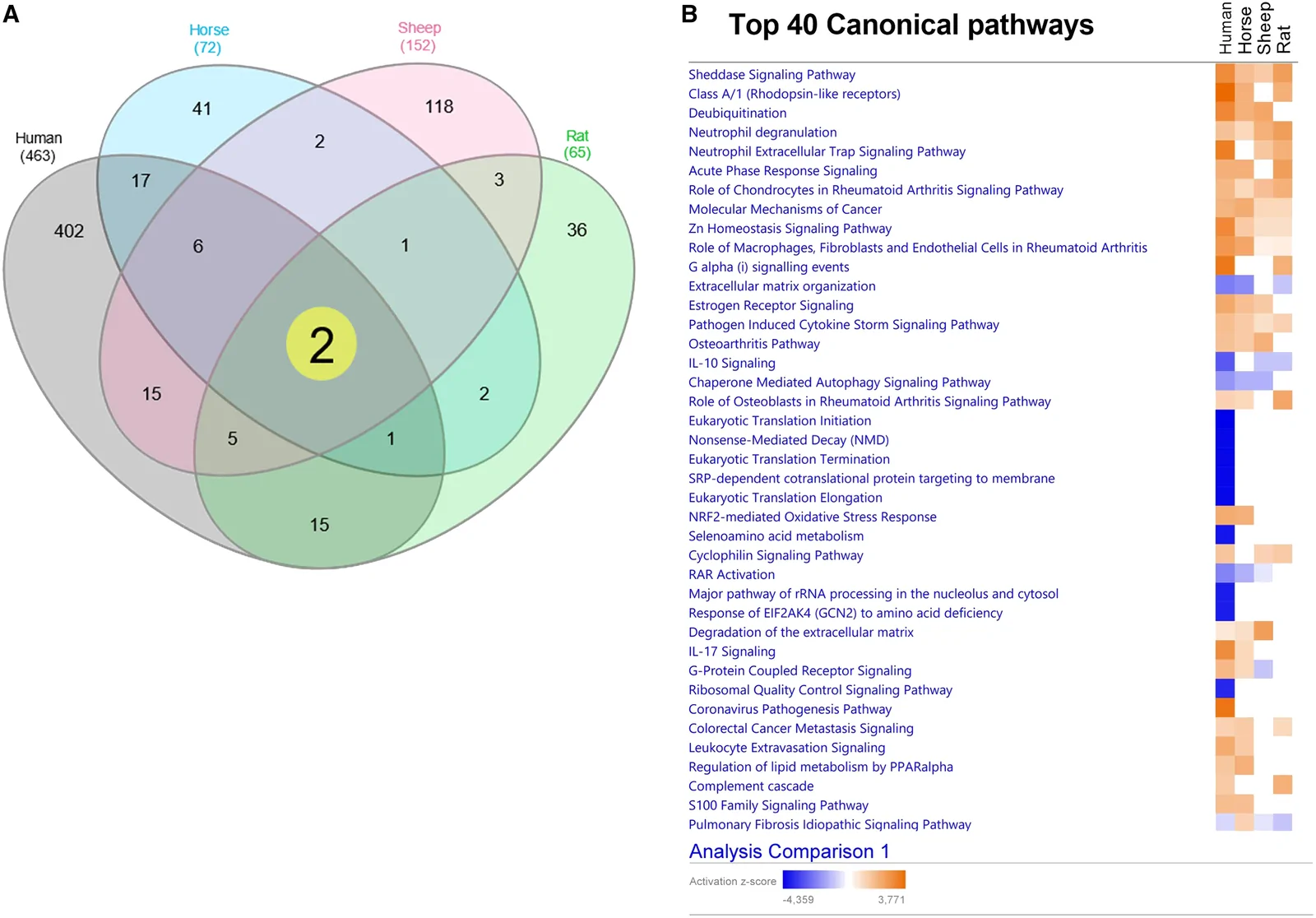
NGS-based comparative analysis of inflammatory responses in chondrocytes across species (A) Venn diagram showing overlap and species-specific distribution of significantly inflammatory differentially expressed genes (DEGs) in human, horse, sheep, and rat. Two genes (*SOD2* and *NFKBIA*) were commonly regulated across all species. (B) Heatmap of the top 40 canonical pathways identified by ingenuity pathway analysis (IPA), ranked by activation *Z* score. Orange indicates pathway activation; blue indicates inhibition. See also Tables S2, S3, S4, S5, S6, and S7.

IPA revealed human chondrocytes regulated 86 canonical pathways (|*z*| ≥ 2), vs. 10 in rat, 9 in horse, 5 in sheep (Table S6). Cross-species comparison identified 9 pathways that were shared between two or more species (Figure 4B; Table S7). Only three pathways were shared by three species (human, horse, and rat): class A/1 (rhodopsin-like receptors), deubiquitination, and acute phase response signaling (Figure 4B). These pathways represent conserved molecular mechanisms through which chondrocytes respond to inflammatory stimuli.

Species-specific canonical pathway activation patterns revealed distinct inflammatory profiles across the studied species (Figure 5). In humans, the central inflammatory drivers were IL-1α, IL-1β, IL-2, IL-17A, IFN-γ, and TNF, with prominent involvement of the OA pathway and hepatic fibrosis/hepatic stellate cell activation. In equine chondrocytes IL-1α, IL-1β, IFN-γ, and TNF also emerged as key nodes, indicating a robust pro-inflammatory response involving hepatic fibrosis/hepatic stellate cell activation (similarly to humans) as well as granulocyte adhesion and diapedesis. In contrast, sheep chondrocytes responded to inflammatory stimulation primarily through rheumatoid arthritis-associated pathways, notably the role of osteoblasts, osteoclasts, and chondrocytes in rheumatoid arthritis and the role of chondrocytes in rheumatoid arthritis signaling pathway. Meanwhile, rat chondrocytes exhibited a unique pattern characterized by both acute inflammatory and regenerative responses, with key pathway engagement including acute phase response signaling and hematoma resolution signaling. Central cytokines in the rat response included IL-1α, IL-1β, IL-6, IL-17A, and TNF, and the transcription factor RELA.

**Figure 5:**
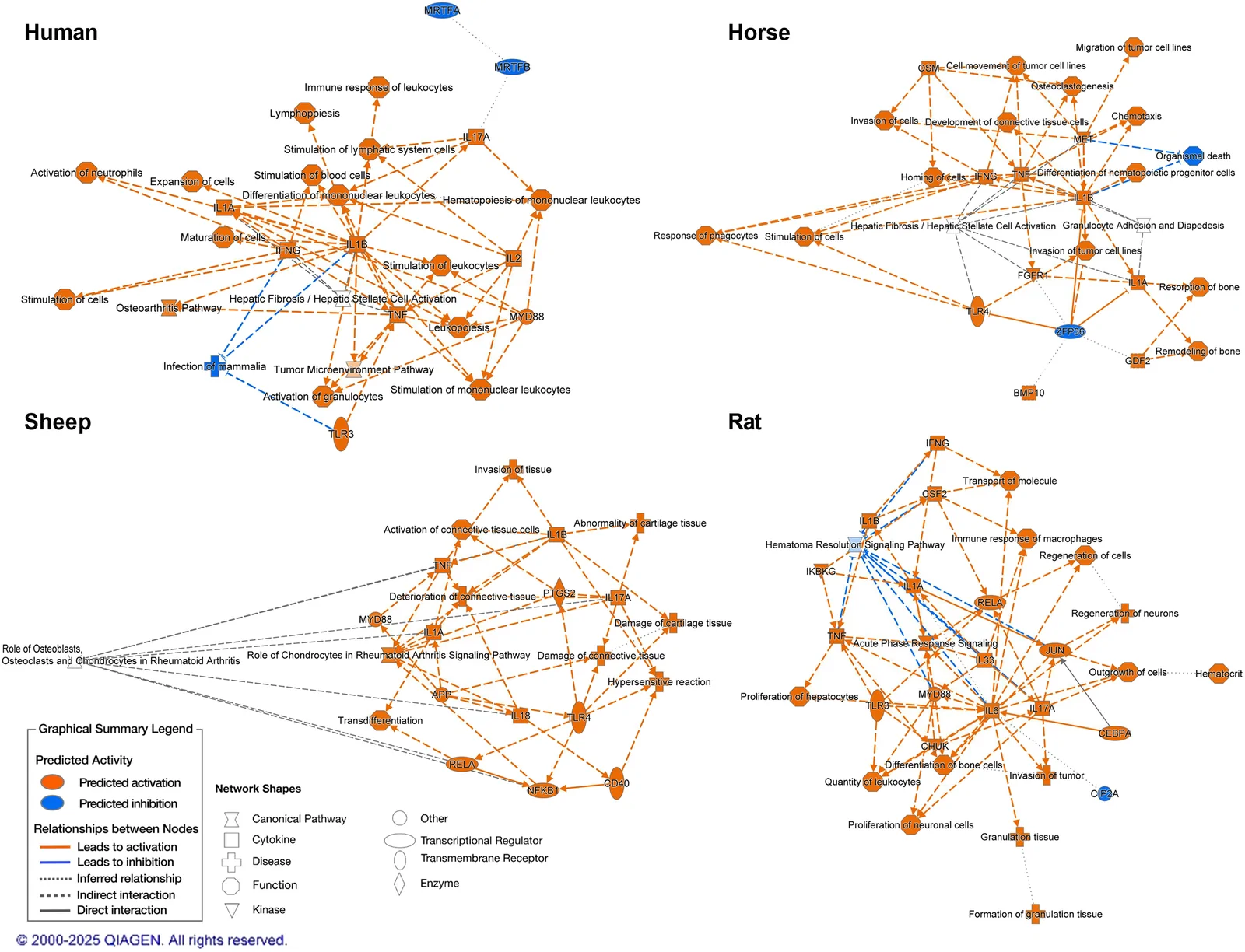
Graphical summary of key canonical pathways activated in inflamed chondrocytes across species Graphs depict networks generated by ingenuity pathway analysis (IPA), highlighting predicted upstream regulators, central molecules, and biological processes. Solid orange lines indicate predicted activation; blue lines indicate inhibition.

### Orthogonal validation of NGS data by quantitative PCR

The direction and magnitude of *IL6*, *MMP1*, *MMP13*, *SOD2*, *TXNRD1*, *COL2A1*, *ACAN*, and *PRG4* expression observed by RNA-seq strongly concordant with qPCR validation (Spearman’s *ρ* = 0.871, *p* < 0.0001), confirming the robustness of the transcriptomic findings (Figure S4; Table S8).

Species, condition (native tissue, vs. healthy 3D vs. inflamed 3D culture) and their interaction all significantly (*p* < 0.05) influenced expression of *IL6*, *MMP1*, *MMP13*, *SOD2*, *TXNRD1*, *COL2A1*, *ACAN*, and *PRG4* (Figures 6A–6H; Table S9). In native human cartilage, for example *COL2A1* levels were lower than in rabbit and rat but higher than in horse, while *ACAN* was reduced relative to all three animal species. In addition, human native also exhibited significantly greater IL-6 expression than horse, higher *TXNRD1* than horse and sheep, elevated *MMP1* compared to sheep, and increased *MMP13* versus rat (Figures 6A–6H; Table S9). Transitioning to 3D culture had distinct, species-specific effects: *IL-6* expression remained unchanged in human and horse but dropped sharply in sheep and rat (both *p* < 0.0001) and rose modestly in rabbit (*p* = 0.0419). *ACAN* declined in all species (*p* < 0.05), and *COL2A1* fell in every species except horse. (Figures 6A–6H; Table S9). Inflammatory stimulation (5 ng/mL IL-1β/TNF-α) markedly upregulated *IL-6* in all species except rabbit, and induced *MMP1* and *MMP13* in all except rabbit (for *MMP1*) and rat (for *MMP13*). Under these conditions, human 3D chondrocytes displayed the highest *IL-6* and *MMP1* levels of any species; *MMP13* expression in inflamed cells did not differ significantly between most species and human, except in horse, where it was significantly upregulated. Inflamed ovine chondrocytes maintained significantly lower *COL2A1* and *PRG4* than human, whereas inflamed rat cells showed higher *ACAN* expression compared to human.

**Figure 6:**
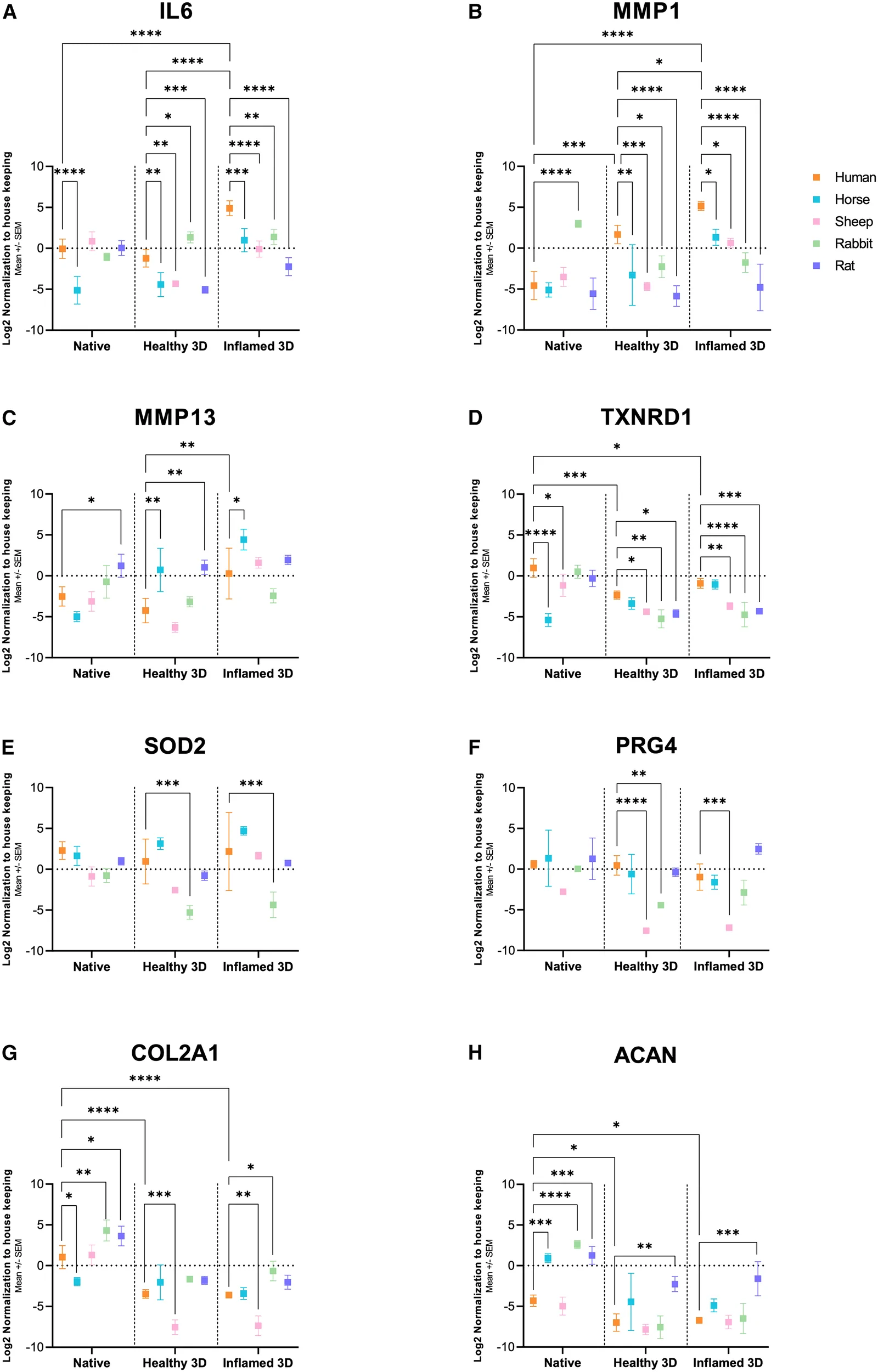
qPCR-based comparative gene expression profiles across species in native tissue, healthy 3D pellets, and inflamed 3D chondrocytes pellets (A–H) Gene expression analysis of inflammatory and extracellular matrix (ECM)-related genes: *IL-6* (A), *MMP1* (B), *MMP13* (C), *TXNRD1* (D), *SOD2* (E), *PRG4* (F),*COL2A1* (G), and *ACAN* (H). Expression levels are shown as log_2_ fold change relative to housekeeping genes (*GAPDH* and *RPLP0*), presented as mean ± SEM. Each image displays gene expression from chondrocytes derived from native tissue (cartilage), healthy, and inflamed (5 ng/mL) 3D pellet culture conditions across five species: human, horse, sheep, rabbit, and rat (*n* = 3 donors). Statistical significance was evaluated using one-way ANOVA, comparing each animal species to the corresponding human condition (native, healthy, and inflamed). ∗*p* < 0.05, ∗∗*p* < 0.01, ∗∗∗*p* < 0.001. See also Table S9.

## Discussion

This study comprehensively evaluated and compared the inflammatory responses of chondrocytes from various commonly used OA animal models (horse, sheep, rabbit, and rat) with human chondrocytes, employing both 2D and 3D culture systems under standardized IL-1β and TNF-α stimulation. Our findings reveal significant interspecies differences across key parameters, including chondrocyte morphology, viability, proliferation, gene expression, and transcriptomic profiles. These results underscore the importance of species-specific considerations in selecting animal models for OA research to enhance translational relevance and adherence to the 3Rs principles.

Murine chondrocytes, particularly susceptible to enzymatic digestion, exhibited apoptosis following passaging and were therefore excluded from downstream analyses. Indeed, due to the notorious difficulty in culturing adult murine articular chondrocytes, owing to their low yield (∼10^5^ cells per mouse), limited proliferation rates, and rapid dedifferentiation in monolayer,^40^ most studies on murine chondrocytes use neonatal or juvenile donors (≤8 weeks), which do not reflect the aged cell population typically associated with OA.^40^ While mice are extensively used as *in vivo* OA models, particularly for genetically modified studies and mechanistic investigations due to their well-characterized genome, ease of genetic manipulation, and high throughput capacity,^41^^,^^42^ their *in vitro* fragility limits assessment of model fidelity and thus the utility of *in vivo* mouse OA models. Differences in joint anatomy (e.g., thinner cartilage in mice,^42^^,^^43^ rapid disease progression in many induced models, and pain phenotyping challenges^42^^,^^43^ further contribute to the translatability challenges of mouse OA models.

Gene expression variability across species highlights distinct regulatory strategies during inflammation. Universal upregulation of *IL-6* and *MMP1* corroborates established OA markers, yet the greater magnitude of induction in human cells emphasizes intrinsic transcriptional sensitivity. The species-specific response might be partly attributed to the use of human recombinant cytokines. The choice to use human cytokines for all species, rather than species-specific cytokines for each species or an alternative non-species-specific inflammatory stimulus such as lipopolysaccharide (LPS), was based on careful deliberation to ensure a standardized inflammatory challenge and enable direct interspecies comparison under identical experimental conditions. Moreover, given that numerous *in vitro* studies of IL-1β and TNF-α-induced chondrocyte inflammation employ human recombinant cytokines,^33^^,^^34^^,^^35^^,^^36^^,^^37^^,^^38^^,^^39^ using human cytokines across all species in this work enhances comparability with the published literature and establishes a cross-species reference framework for future investigations. While the use of human cytokines may influence receptor-binding affinity and cross-reactivity across species, using species-specific cytokines would not have eliminated cross-species variability and could have introduced additional confounders, as cytokine purity, bioactivity, and receptor-binding kinetics vary across commercial preparations and are not equivalently characterized for all veterinary species. LPS, although commonly used as an inflammatory stimulus, triggers a broad TLR4-mediated innate immune activation that does not accurately model the cytokine-driven, low-grade inflammation characteristic of OA. In contrast, IL-1β and TNF-α stimulation reflects the canonical OA-associated inflammatory axis. Cytokines such as IL-1β (β-trefoil family) and TNF-α (β-sandwich family), characterized by their β-sheet based folds, are highly conserved and generally exhibit significant biological cross-reactivity across species.^29^^,^^31^^,^^44^^,^^45^^,^^46^ An established threshold for cytokine cross-reactivity on heterologous cells is a minimum of 60% amino acid identity.^29^^,^^44^^,^^45^^,^^46^ In this study, Basic Local Alignment Search Tool (BLAST) analysis revealed high sequence homology between human IL-1β and TNF-α and the included model species by (IL-1β: 60% (sheep)-74% (rabbit) relative to human; TNF-α: 78% (rat)-87% (horse) relative to human, Figure S2). This suggests cross-reactivity between human-derived cytokines and the corresponding receptors in the model species, which has been experimentally confirmed for all included species.^33^^,^^34^^,^^35^^,^^36^ Therefore, rather than by cytokine-receptor mismatch, the observed species-specific responses may be profoundly influenced by species-specific receptor repertoires, mechanisms of post-translational receptor shedding, and intracellular adapter usage,^44^^,^^47^^,^^48^^,^^49^^,^^50^^,^^51^ all of which have evolved distinct characteristics across different animal lineages.

Cross-species gene expression and transcriptomic analyses revealed that superficial similarities in limited qPCR marker panels can obscure significant species-specific differences in inflammatory signaling at the pathway level, potentially leading to misleading conclusions. For example, equine chondrocytes differentially expressed oxidative stress markers (*SOD2*, *TXNRD1*) and ECM genes (*COL2A1*, *ACAN*, and *PRG4*) at levels comparable to human chondrocytes, despite substantial differences in their broader transcriptomic response. This observation is consistent with previous studies in humans and rodents demonstrating that agreement at the level of individual genes does not necessarily indicate conservation of regulatory networks or pathway dynamics.^52^ Conversely, differences in individual gene orthology or expression profiles do not necessarily equate to functional divergence. Mice, for instance, lack direct orthologs of human *MMP1* and *IL-8* (*CXCL8*), yet mount functionally analogous responses via alternative genes such as *MMP3* and *MMP13* for matrix degradation and *CXCL6* (Granulocyte Chemotactic Protein 2; GCP-2) for neutrophil recruitment.^53^^,^^54^^,^^55^ These examples underscore that individual gene-level data—whether concordant or discordant—are insufficient to predict cellular behavior and highlight the importance of transcriptome-wide analysis for accurately assessing the molecular fidelity of preclinical models.

In this study, comparative transcriptomic profiling of 3D-cultured chondrocytes revealed substantial quantitative differences in DEGs with human chondrocytes exhibiting 463 DEGs, whereas horse, sheep, rat, and rabbit showed only 72, 152, 65, and 0 DEGs, respectively, necessitating the exclusion of rabbits from downstream analysis. Among the DEGs, only *SOD2* and *NFKBIA* were differentially expressed in all species and six DEGs (*BIRC3*, *MMP1*, *MMP13*, *FTH1*, *PDGFRA*, and *AEBP1*) were shared between humans, horses, and sheep. The shared differential expression of downstream targets across species reinforces the IL-1-NF-κB-MMP axis and oxidative stress response as central inflammatory pathways conserved in chondrocytes from humans, rodents, and large-animal models, corroborating previous studies that demonstrated similar regulation of these pathways in different mammalian species.^56^^,^^57^^,^^58^^,^^59^ In addition, inflammatory stimulation induced a shared upregulation of key inflammatory and stress-related signaling pathways in chondrocytes from all four species, including Sheddase signaling,^60^ neutrophil degranulation, role of chondrocytes in rheumatoid arthritis signaling, molecular mechanisms of cancer, zinc homeostasis signaling,^61^ Role of macrophages, fibroblasts, and endothelial cells in rheumatoid arthritis, and pathogen-induced cytokine storm signaling, although the magnitude of pathway activation varied between species. This conserved transcriptional response suggests a common, evolutionarily preserved multifaceted inflammatory and stress response in chondrocytes that may contribute to cartilage degeneration across species.

Nevertheless, crucial divergences in inflammatory signaling between species emerged. Key pro-inflammatory pathways, including “neutrophil extracellular trap signaling pathway” and “interleukin-1 family signaling” were strongly activated in human chondrocytes but not in equine, ovine, and rat. Conversely, “NF-κB signaling” showed activation in equine but not in human cells, implying distinct regulatory dynamics between species. Furthermore, the “osteoarthritis pathway” itself was activated in human, equine, and ovine but notably not in rat, suggesting that the rat model, under this inflammatory stimulus, may not fully recapitulate the canonical OA-related gene expression profile observed in larger animals and humans. The varied activation patterns of key inflammatory pathways even under controlled standardized cytokine stimulation, demonstrate that the precise molecular cascade driving chondrocyte inflammation differs between species. These molecular divergences, coupled with known physiological and biomechanical differences, underscore that no single animal model perfectly mirrors the intricate, multifactorial molecular landscape of human OA. This necessitates careful model selection and species-specific research to ensure translational relevance for developing effective OA therapies, whose efficacy may depend on the specific inflammatory pathways active in the target species. To further explore the interspecies similarities and differences in chondrocytes’ inflammatory responses, additional studies utilizing alternative inflammation induction methods are warranted.

The interspecies differences in the IL-1β/TNF-α-induced inflammatory responses in chondrocytes across species have broad translational significance. Characterizing species-specific inflammatory signatures to key OA cytokines is essential for the evidence-based selection of animal models based on their mimicry of specific molecular endotypes of OA. Identifying species whose transcriptomic and inflammatory profiles most closely reflect those of humans can improve the predictive power of preclinical models, decrease translational attrition, and advance the 3R by reducing unnecessary animal use.^6^^,^^62^^,^^63^^,^^64^ Beyond their translational implications, these findings are also relevant for veterinary medicine. The pronounced species-specific transcriptomic responses revealed by RNA-seq indicate that OA biomarkers or therapeutics developed for human use may require adaptation for veterinary patients.

In summary, inflammatory mechanisms are central to the pathogenesis of diverse OA endotypes.^65^^,^^66^ Therefore, an animal model’s inflammatory response must closely emulate that of humans to be genuinely suitable for OA research and therapeutic development.

This study highlights the complexity and species-specific nature of chondrocyte inflammatory responses. No single animal model fully mirrors the multifaceted inflammatory profile observed in human OA. As OA therapies become increasingly precise and mechanistically targeted, the limitations of directly extrapolating data from animals to humans are becoming more apparent and consequential. To improve preclinical efficacy and reduce translational attrition, a paradigm shift is needed toward more strategic and evidence-based selection of animal models—guided by transcriptomic and functional fidelity rather than structural resemblance alone. The integration of cross-species transcriptomic analysis provides a powerful tool to benchmark the translational relevance of animal models and to identify species with molecular profiles that most closely align with human disease biology. Where cross-species mimicry is lacking or insufficient, human 3D *in vitro* models may offer the most reliable and ethically sound approach for investigating specific mechanisms. Together, these strategies can enhance model validity, uphold the 3Rs’ principles, and ultimately accelerate the development of effective, targeted-OA therapies for both human and veterinary patients.

### Limitations of the study

There are few limitations to this study. First, the human cartilage samples were obtained from patients undergoing arthroplasty and therefore may not represent completely healthy tissue. This could influence the basal gene expression profile and responsiveness of human chondrocytes compared to those from other species. Second, for the next-generation sequencing (NGS) data, the annotation and identification of genes were less complete in some species, which may have affected the accuracy of cross-species comparisons, particularly with human data. These factors should be considered when interpreting the comparative transcriptomic analyses. Third, only female-derived samples were included in this study, reflecting the higher prevalence and risk of OA observed in women, particularly with increasing age.^1^ Consequently, the potential influence of sex on chondrocyte responses to inflammatory stimuli could not be evaluated. The findings may therefore not be fully generalizable to male-derived cells, and future studies including both sexes will be necessary to determine whether sex-related differences influence the species-specific responses observed in this study.

## Resource availability

### Lead contact

Requests for further information and resources should be directed to and will be fulfilled by the lead contact, Florien Jenner (florien.jenner@vetmeduni.ac.at).

### Materials availability

This study did not generate new unique reagents.

### Data and code availability

• All data generated and analyzed during this study are included in this published article and its supplemental information files or are available in the NCBI sequence read archive (SRA) under the accession numbers PRJNA1330137 (human data) and PRJNA1330577 (other species) as of the date of publication.
• This paper does not report original code.
• Any additional information required to reanalyze the data reported in this paper is available from the lead contact upon request.

## Acknowledgments

We would like to thank Maik Dahlhoff, Institute of In-vivo and In-vitro Models, University of Veterinary Medicine, Vienna and Patrick Bilic, Center for Biomedical Science and Translational Surgery, Medical University, Vienna for providing material. Next-generation sequencing and initial data analysis was performed by the Biomedical Sequencing Facility at CeMM Research Center for Molecular Medicine of the Austrian Academy of Sciences. We also gratefully acknowledge the support of the Ernst Mach 10.13039/100016234ASEA-UNINET PhD scholarship from Austria's Agency for Education and Internationalization (OeAD), awarded to the A.K.

## Author contributions

A.K., data analysis and interpretation, and manuscript preparation; M.-B.A., data analysis and interpretation; K.T., data analysis and interpretation, and manuscript preparation; S.G., data acquisition, analysis, and interpretation; M.R., data acquisition; I.G., data acquisition and analysis; F.J., study conception and design, data analysis and interpretation, manuscript preparation. All authors reviewed the manuscript.

## Declaration of interests

The authors declare no competing interests.

## Declaration of generative AI and AI-assisted technologies in the writing process

The authors used ChatGPT 4o for linguistic refinement. After using this tool, the authors reviewed and edited the content as needed and take full responsibility for the content of the publication.

## STAR★Methods

### Key resources table

**Table:** 

REAGENT or RESOURCE	SOURCE	IDENTIFIER
**Biological samples**		

Human primary chondrocytes	ViBiMeD Biobank, Medical University, Vienna, Vienna, Austria	N/A
Equine, ovine, rats, and mice C57BL/6 strain (for cartilage sample)	University of veterinary medicine, Vienna, Vienna, Austria	Mice: RRID:IMSR_JAX:000664
Rabbit (for cartilage sample)	Center for Biomedical Science and Translational Surgery, Medical University, Vienna, Austria	N/A

**Chemicals, peptides, and recombinant proteins**		

Human interleukin-1b (IL-1b)	ImmunoTools GmbH	Cat# 11340013
Human tumor necrosis factor-a (TNF-a)	ImmunoTools GmbH	Cat# 11343013
Collagenase II	Sigma-Aldrich	Cat# C2674-1G; RRID:SCR_008988
Pronase®	Sigma-Aldrich	Cat# 537088-5KU; RRID:SCR_008988
Phalloidin	Sigma-Aldrich	49409; Cat# 49409-10NMOL; RRID:SCR_008988
DAPI	Invitrogen by Thermo Fisher Scientific	Cat# 10374168

**Critical commercial assays**		

CellTiter 96® AQueous One Solution Cell Proliferation Assay (MTS/MTT assay)	Promega	Cat# G3580; RRID:SCR_006724
Caspase-3/7 Green ReadyProbes™ Reagent	Invitrogen by Thermo Fisher Scientific	Cat# 13599180
Senescence Assay Kit	Fluorometric format, Cell Biolabs	Cat# CBA-231-CB
Qubit Protein Assay Kit	Invitrogen	Cat# 10788713
QuantSeq 3′ mRNA-Seq V2 Library Prep Kit with the UDI FWD kit	Lexogen GmbH	N/A

**Deposited data**		

All analyzed data	This paper	N/A
Human RNA sequencing data	This paper	PRJNA1330137
Animal RNA sequencing data	This paper	PRJNA1330577

**Oligonucleotides**		

See Table S10 for all primers	This paper	N/A

**Software and algorithms**		

ImageJ software	NIH	https://fiji.sc/
IncuCyte® software analysis	Sartorius	RRID:SCR_003935; https://www.sartorius.com/en/product
STAR (v2.7.11b)	Dobin, A et al.^67^	https://www.ncbi.nlm.nih.gov/pubmed/23104886
DESeq2 in R (v4.4.2)	N/A	N/A
Venn diagrams	N/A	www.interactivenn.net
Ingenuity Pathway Analysis	Qiagen	https://digitalinsights.qiagen.com/products-overview/discovery-insights-portfolio/analysis-and-visualization/qiagen-ipa/; RRID:SCR_008539
GraphPad Prism 9.5.1	GraphPad Software	https://www.graphpad.com/; RRID:SCR_000306
Bioconductor DESeq2 (1.46.0) package	Love MI, Huber W, Anders S (2014)	https://doi.org/10.1186/s13059-014-0550-8 https://bioconductor.org/packages/release/bioc/html/DESeq2.html

**Other**		

Human and mouse reference genomes	GENCODE	https://doi.org/10.1093/nar/gkaa1087
Equine, ovine, rabbit reference genomes	Ensembl	N/A

### Experimental model and study participant details

Articular cartilage was harvested from female, middle-aged donors of six species (*n* = 3 biological replicates per species): humans (*Homo sapiens*, aged 52–58 years), horses (*Equus caballus*, aged 11–19 years), sheep (*Ovis aries*, aged 2–5 years), rabbits (*Oryctolagus cuniculus*, aged 5–6 months), rats (*Rattus norvegicus domestica*, aged 5–6 months), and mice (*Mus musculus*, C57BL/6 strain, aged 2–4 months).

Human articular cartilage was collected from total knee arthroplasty patients with ethical approval of the ethics committee of the Medical University of Vienna; ViBiMeD Biobank, Ethics Approval No. 1822/2017 and 1034/2020) and informed consent by the patients. For all other species, tissues were harvested post-mortem from animals euthanized at the University of Veterinary Medicine for reasons unrelated to this study; where animals were client-owned, tissue use was subject to owner consent as per institutional guidelines. Animal handling and euthanasia were carried out according to the relevant veterinary, institutional, and regulatory standards. Per institutional policy (University of Veterinary Medicine Vienna), tissue from animals euthanized for reasons unrelated to a given study is classified as post-mortem biowaste, and hence its use in post-mortem *in vitro* work, as in the present study, does not require additional ethics approval.

The number of biological replicates (*n* = 3) is based on a power analysis conducted using data from pilot experiments in which inflammatory cytokine dosages were titrated to achieve a consistent inflammatory response across chondrocytes from different species, as determined by qPCR. Following stimulation with 5 ng/mL IL-1β and TNF-α, the cytokine concentration selected for downstream experiments, IL-6 expression (mean ± SD fold change to healthy controls: Human 7.86 ± 0.46; Equine 5.03 ± 1.79; Murine 2.72 ± 0.71; Ovine 1.74 ± 0.38; Rabbit 3.79 ± 0.58) was used for a one-way ANOVA power simulation (α = 0.05, 10,000 iterations). The analysis indicated that *n* = 3 biological replicates per species achieved statistical power > 0.99 for detecting inter-species differences in IL-6 expression.

### Method details

#### Articular cartilage harvest and chondrocyte isolation

Cartilage was dissected from femoral condyles in humans, horses, sheep, and rabbits, and from femoral heads in rats and mice. Specimens showing macroscopic osteoarthritic changes were excluded. Immediately after harvesting, cartilage specimens were placed in phosphate-buffered saline (PBS +/+; Mg2+/Ca2+) supplemented with 1% penicillin-streptomycin (Pen/Strep) (Sigma-Aldrich, USA), cartilage was minced and digested in 0.1 mg/mL collagenase II (Sigma-Aldrich, USA) at 37 °C with gentle agitation for 2–3 h. For mouse, rat, and rabbit samples, a 30-minute predigestion with 0.5% Pronase® (Sigma-Aldrich, German) was performed prior to collagenase treatment to enhance chondrocyte yield. Digestion was stopped by adding an equal volume of Dulbecco's Modified Eagle Medium (DMEM) (Gibco, Thermo Fisher Scientific, USA), supplemented with 10% fetal bovine serum (FBS) (Capricorn Scientific, FBS-12A, Germany), 1% Pen/Strep, and 1% amphotericin B (Life Technologies Limited, UK), hereafter referred to as the “standard culture medium”. The resulting cell suspension was filtered through a 100 μM cell strainer (Greiner Bio One, Austria) and centrifuged at 540x g for 5 min. The cell pellet was washed twice with PBS and then seeded into standard culture medium in appropriately sized flasks: T25 flask for mouse cells, T75 flask for rat cells and T175 flasks for the other species. Cells were cultured at 37 °C with 5% CO_2_. Medium was changed every 3 days until 90–100% confluence, then cells were cryopreserved in 90% FBS and 10% DMSO at −150°C.

#### Morphological analysis

Chondrocytes cell morphology was evaluated using Phalloidin-DAPI staining. All experiments used passage 4 chondrocytes except murine cells (passage 1). Cells were culture to ∼50% confluence. After fixation (4% paraformaldehyde, 10 min), cells were permeabilized (0.2% Triton X 100, 20 min) and blocked (5% goat serum (G9023-10ML, Sigma-Aldrich, USA), 30 min). Actin filaments were stained with Phalloidin (Alexa Fluor 488 (49409, Sigma-Aldrich, USA), 20 μL/mL in 1% goat serum; 30 min), and nuclei with DAPI (Invitrogen, USA) (250 ng/mL; 5 min). Cells were imaged using the EVOS imaging system (10×, 40×) (EVOS FL AUTO; life technologies, United States), and cell area, circularity, and nuclear-to-cytoplasmic ratio were quantified using Image J software (NIH, USA).

#### Additional assays for murine chondrocytes

##### SA-β-galactocidase

Chondrocytes were seeded at an initial density of 1,000 cells per well in 96-well plate and cultured at 37°C, 5% CO2 for 96 hours. Then cells were washed twice with ice-cold PBS and lysed with 60 μL of cell lysis buffer from the Cellular Senescence Assay Kit (Fluorometric format, Cell Biolabs, USA) for 10 minutes at 4°C. The whole-cell lysates were collected into Eppendorf tubes and centrifuged at 13,000g for 15 minutes at 4°C. Total protein concentrations were determined using the Qubit Protein Assay Kit (ThermoFisher scientific, USA) and measured with the Qubit 4 Fluorometer (ThermoFisher scientific, Singapore, shipped from Germany) following the manufacturer’s instructions. After protein quantification, the assay buffer (Cell Biolabs, Inc., CBA-231, San Diego, USA) was added to the cell lysates in a 1:1 ratio and incubated in the dark at 37°C for 3 hours. The reaction was then stopped by adding 120 μL of Stop Solution per 30 μL of reaction mixture. Finally, fluorescence was measured using a plate reader at 360 nm excitation and 465 nm emission, with SA-β-Gal activity normalized to total protein concentrations.

##### Caspase 3/7 activity assessment

To assess cell apoptosis, the Caspase-3/7 Green ReadyProbes™ Reagent (Invitrogen by Thermo Fisher Scientific. USA) was used according to the manufacturer’s instructions. In brief, chondrocytes were seeded at 1,000 cells/well in a 96-well plate and cultured at 37°C with 5% CO_2_. After 24 hours, the medium containing Caspase-3/7 Green ReadyProbes™ Reagent was refreshed and observed using the IncuCyte® S3 Kinetic Live-Cell Imaging System, which captured images at 2-hour intervals over 24 hours. Apoptotic cell number were calculated based on fluorescence intensity increases in the cell with caspase 3/7 activity by using the IncuCyte® software analysis.

#### Inflammation dose titration

To determine the inflammatory stimulation necessary to induce a consistent inflammatory response in all species, chondrocytes were treated with recombinant human IL-1ß and TNF-α (ImmunoTools GmbH, Germany) at 1, 2.5, 5 or 10 ng/mL for 72h. Owing to the lack of species-specific cytokines for certain models, human recombinant IL-1ß and TNF-α were employed, as their ability to effectively induce inflammation in chondrocytes of all five species had been previously established.^33^^,^^34^^,^^35^^,^^36^^,^^37^^,^^38^^,^^39^ Cells were seeded in 2D (15000 cells/well in 12-well plates), allowed to adhere 24 h (T0), then exposed to cytokines for 24 h (T24). Then media were refreshed (healthy: fresh standard culture medium, inflamed: fresh medium with IL-1ß and TNF-α) and cells were cultured for another 48 hours (T72). Expression of *IL6*, *MMP1*, *MMP3*, and *MMP13* was assessed by RT-qPCR, and viability by MTT assay.

#### Cell viability - MTT asssay

At T72, MTT reagent (Promega, USA) was added 1:1 to culture medium and incubated 2–3 h at 37 °C. Absorbance was measured at 595 nm using a plate reader (Varioskan LUX, Thermo Scientific, USA). For each species, inflamed-to-healthy viability ratios at each cytokine concentration were calculated.

#### 2D vs. 3D inflammatory response

A cytokine dose of 5 ng/mL (each IL-1ß and TNF-α) was selected for subsequent experiments. For 2D, passage 4 cells were seeded and cultured as described above. For 3D, 300,000 cells were pelleted (330× g, 5 min) and cultured in 15 ml falcon tube in 1 ml standard culture medium for 96 h to form spheroids.

Inflammatory medium was added 24 hours after seeding in 2D cultures and 96 hours after seeding in 3D cultures (T0). After 24 hours of exposure (T24), the medium was refreshed (healthy: fresh standard culture medium, inflamed: fresh inflammatory medium). Samples were harvested at 72 hours (T72) for RT-qPCR (2D and 3D samples) and next-generation sequencing (NGS, 3D samples). In addition, the inflammatory response of monolayer cultured chondrocytes was assessed using proliferation and wound healing (scratch) assays.

#### Proliferation assay

Chondrocyte growth was monitored in IncuCyte® S3 live-cell imager (Sartorius, Germany), with phase-contrast images captured every 2 h for 48 h. Confluence data were extracted using IncuCyte® software. Fold-change in confluence at 48 h (T72) was calculated for healthy vs. inflamed conditions within and across species.

#### Wound healing (scratch) assay

Cells were seeded (5,000 cells/well) in IncuCyte® ImageLock 96 well plates (Sartorius, Germany). At T24 (after inflammatory treatment), uniform scratches were made using the IncuCyte® Woundmaker (Sartorius, Germany). Images were acquired every 2 h for 48 h (T24–T72). Wound closure (%) was quantified over this 48-hour period using the IncuCyte® S3 system (10x magnification) and compared across conditions (healthy vs. inflamed) and species. Measurements were based on background cell density of the wound at the initial time point, and for changes in both the density of the cell (non-wound) and the wound region (relative wound density; RWD). RWD was calculated by the IncuCyte® S3 software using the following formula.

$$\%\text{RWD}\left(t\right)=100\times \frac{\left(w\left(t\right)-w\left(0\right)\right)}{\left(c\left(t\right)-w\left(0\right)\right)}$$

Where w(t) is the density of the wound region at time (t) and c(t) is the density of the cell region at time (t). RWD is a validated metric of the IncuCyte platform that normalises wound closure for concurrent changes in cell density outside the wound area, thereby isolating migratory capacity from proliferative contributions^68^^,^^69^^,^^70^^,^^71^ — a distinction of particular relevance in the present study given the significant interspecies differences in proliferation rate.

#### RNA extraction

Native cartilage, as well as 2D and 3D cultures harvested at T72, were lysed in TRIzol (Invitrogen, Thermo Scientific, Germany) supplemented with 1% ß-mercaptoethanol (Sigma-Aldrich, USA). RNA was extracted using a standard TRIzol–chloroform protocol (detailed protocol: https://protocols.io/view/rna-isolation-dnhm5b46.html). RNA concentration was quantified using a NanoPhotometer N60 Touch (Implen, Germany).

#### Transcriptomic profiling of chondrocyte pellets via 3′ RNA-Sequencing (NGS)

Total RNA of chondrocyte pellets was processed using the QuantSeq 3′ mRNA-Seq V2 kit (Lexogen) and sequenced on an Illumina NovaSeq 6000 (100 bp, single-end). Reads were mapped to species-specific reference genomes (GENCODE for human and mouse; Ensembl for others) using STAR (v2.7.11b) with ENCODE-recommended settings.^67^^,^^72^ Gene-level counts were generated with Bioconductor’s Genomic Alignments, and differential expression analysis (healthy vs. inflamed) was performed with DESeq2 in R (v4.4.2). Differentially expressed genes (DEGs) were filtered by FDR < 0.1 and |fold change| ≥ 0.25, then analyzed via Ingenuity Pathway Analysis (IPA, Qiagen) to identify enriched pathways, upstream regulators, and gene networks. Overlapping and species-specific DEGs were visualized using Venn diagrams (www.interactivenn.net, accessed April 2025).

#### Orthogonal validation of NGS data by quantitative PCR (qPCR)

RNA samples at a concentration of 1 ng/μL were used for RT-qPCR analysis (detailed protocol: https://protocols.io/view/rt-qpcr-dnep5bdn.html). A targeted panel of 8 key inflammatory and extracellular matrix (ECM)-related genes, including *IL6*, *MMP1*, *MMP13*, superoxide dismutase 2 (*SOD2*), thioredoxin reductase 1 (*TXNRD1*), collagen type II alpha 1 (*COL2A1*), aggrecan (*ACAN*), and proteoglycan 4 (*PRG4*), plus two housekeeping genes glyceraldehyde-3-phosphate dehydrogenase (*GAPDH*) and acidic ribosomal phosphoprotein P0 (*RPLP0*) was used to orthogonally validate NGS results and to compare expression in native cartilage for each species. Relative expression levels were calculated using the 2ˆ–ΔCt method, normalized to *GAPDH* and *RPLP0*. Expression levels in native tissue and cultured chondrocytes (both healthy and inflamed) from horse, sheep, rabbit, and rat were compared across species under the same condition (native tissue, healthy, and inflamed) and within each species across different conditions, to confirm conserved and species-specific expression patterns observed in the NGS dataset.

### Quantification and statistical analysis

Statistical analyses for all non–NGS datasets (incl. qPCR, proliferation, wound healing, morphological parameters) were performed using GraphPad Prism 9.5.1 (GraphPad Software, San Diego, CA, USA). Data distribution was evaluated using the D’Agostino–Pearson omnibus normality test. As the datasets met the assumption of normality, analysis of variance (ANOVA) was applied, followed by either Tukey’s post hoc test for ordinary one-way ANOVA and two-way ANOVA or Dunnett’s correction for repeated-measures ANOVA with Geisser–Greenhouse adjustment where appropriate.

Data are presented as mean ± SEM. For all experiments, *n* = 3 biological replicates, with n representing independent biological samples/replicates. The exact statistical comparisons and tests applied to individual experiments are indicated in the corresponding figure legends.

To evaluate concordance between NGS and qPCR expression estimates, Spearman’s rank correlation (ρ) was computed across matched samples. Spearman’s ρ was used as the primary metric because the common nonlinearity between NGS and qPCR data, arising from differences in their dynamic range, makes rank-based correlation, which captures consistency in direction and relative magnitude rather than strict linearity, more appropriate for validating NGS results.

A two-sided *p*-value < 0.05 was considered statistically significant for all analyses.

## Supplemental information

Document S1. Figures S1–S4 and Tables S1, S7, S8, and S10

Table S2. Significant differentially expressed genes in human chondrocytes, related to Figure 4 This table presents the list of 463 significant DEGs detected in human chondrocytes 3D pellet culture. The summary includes genes that are uniquely regulated in human samples as well as DEGs shared across other species. Significance was determined based on adjusted *p* values <0.1 and |fold change| ≥ 0.25.

Table S3. Significant differentially expressed genes in horse chondrocytes, related to Figure 4 This table presents the list of 72 significant DEGs detected in horse chondrocytes 3D pellet culture. The summary includes genes that are uniquely regulated in horse samples as well as DEGs shared across other species. Significance was determined based on adjusted *p* values <0.1 and |fold change| ≥ 0.25.

Table S4. Significant differentially expressed genes in sheep chondrocytes, related to Figure 4 This table presents the list of 152 significant DEGs detected in sheep chondrocytes 3D pellet culture. The summary includes genes that are uniquely regulated in sheep samples as well as DEGs shared across other species. Significance was determined based on adjusted *p* values <0.1 and |fold change| ≥ 0.25.

Table S5. Significant differentially expressed genes in rat chondrocytes, related to Figure 4 This table presents the list of 65 significant DEGs detected in rat chondrocytes 3D pellet culture. The summary includes genes that are uniquely regulated in rat samples as well as DEGs shared across other species. Significance was determined based on adjusted *p* values <0.1 and |fold change| ≥ 0.25.

Table S6. Significant canonical pathways across all species, related to Figure 4 The table summarizes significant canonical pathways, and corresponding *Z* scores identified by ingenuity pathway analysis (IPA) across human, horse, sheep, and rat datasets. Pathways are listed based on their statistical significance (|*z*| ≥ 2) and predicted activation state.

Table S9. Summary of multiple comparisons between species, related to Figure 6 Table S9 presents the results of Tukey’s multiple comparisons test assessing gene expression differences among five species—human, horse, sheep, rabbit, and rat—under three experimental conditions: native tissue, healthy 3D culture, and inflamed 3D culture. For each condition, pairwise comparisons were conducted for the following genes: *IL-6*, *MMP1*, *MMP13*, *SOD2*, *TXNRD1*, *COL2A1*, *ACAN*, and *PRG4*. (significant adjusted *p* value <0.05).
