# Supplementary material for: Species-specific inflammatory responses of chondrocytes: Toward evidence-based model selection in OA research
Source: iScience. 2026 Sep 22;29(10):117710. doi: 10.1016/j.isci.2026.117710 (PMC13657218; doi:10.1016/j.isci.2026.117710)
Supplement: Supplementary file 1 [file mmc1.pdf]

iScience, Volume *29*

Supplemental information

Species-specific inflammatory responses of chondrocytes: Toward evidence-based model selection in OA research

Angkana Kidtiwong, Maria-Belen Arteaga, Karyna Tarasova, Sinan Gültekin, Mario Rothbauer, Iris Gerner, and Florien Jenner

Supplementary materials

Supplementary Figures

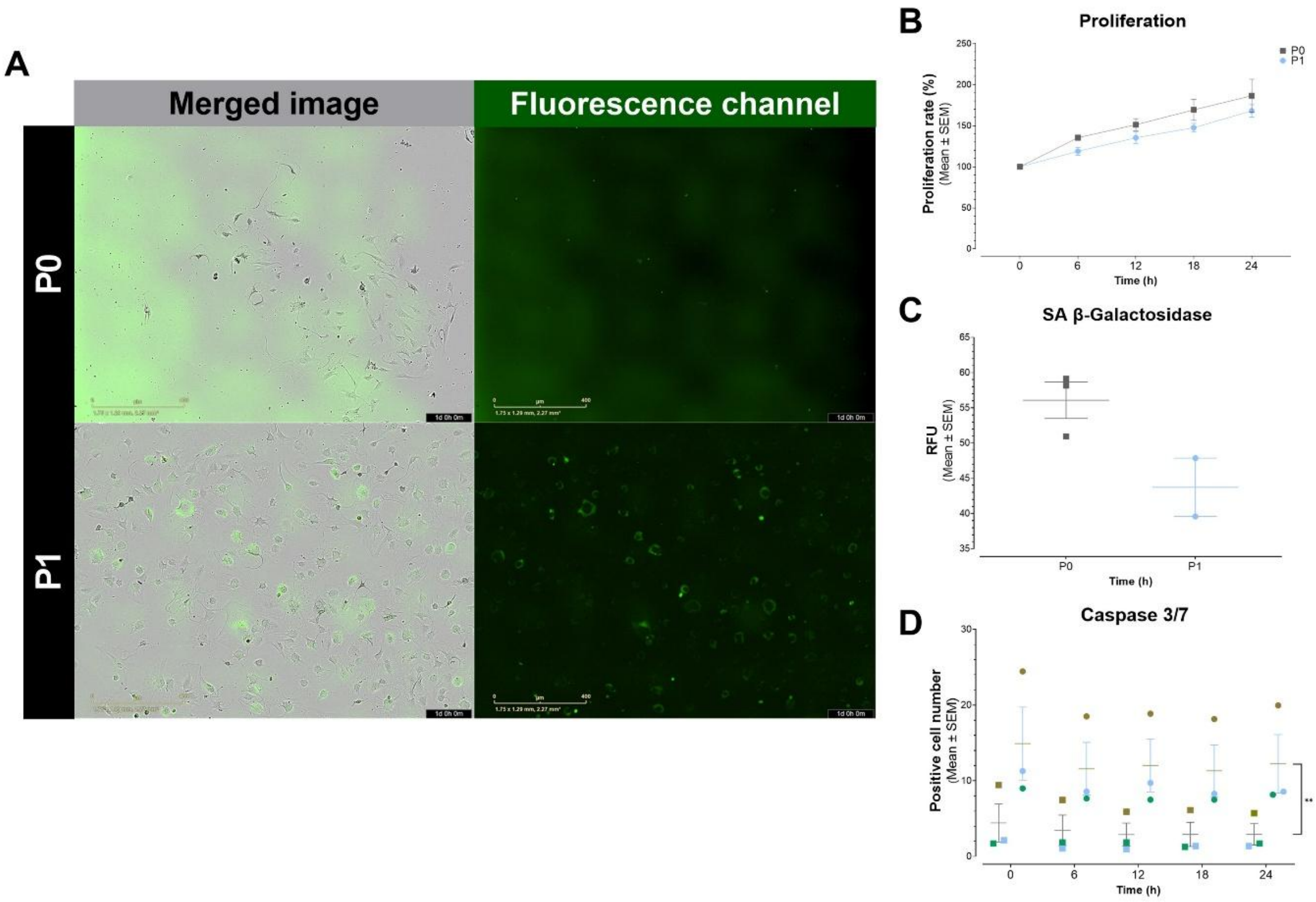

Figure S1: Comparison of mouse chondrocyte proliferation, senescence-associated beta-galactosidase (SA-β-galactosidase) and caspase 3/7 activity. Related to results 'murine chondrocyte apoptosis' section. Data are presented as mean ± SEM from three biological donors (n = 3)

(A) Representative merged (phase contrast + fluorescence) and fluorescence channel images of P0 and P1 murine chondrocytes at 24 hours. Green fluorescence indicates caspase 3/7 activity. P1 cells show visibly increased caspase 3/7-positive fluorescent signal compared to P0, consistent with elevated apoptotic activity following trypsinization. (Representative caspase-3/7 images from one of the three mice included in the experiment are shown)

(B) Proliferation of P0 and P1 chondrocytes over 24 hours was analyzed using two-way ANOVA. No statistically significant difference in proliferation was observed between passages.

(C) SA-β-galactosidase activity in P0 and P1 chondrocytes at 24 hours (Individual data points are shown) was analysed by unpaired t-test. No significant difference in SA-β-galactosidase activity was detected between passages.

(D) Caspase 3/7-positive cell number over 24 hours in P0 and P1 chondrocytes (Individual data points are shown) was analysed by unpaired t-test. Caspase 3/7 activity was significantly elevated in P1 cells at 24 hours compared to P0 (** $p < 0.01$), indicating increased apoptotic activity following passaging.

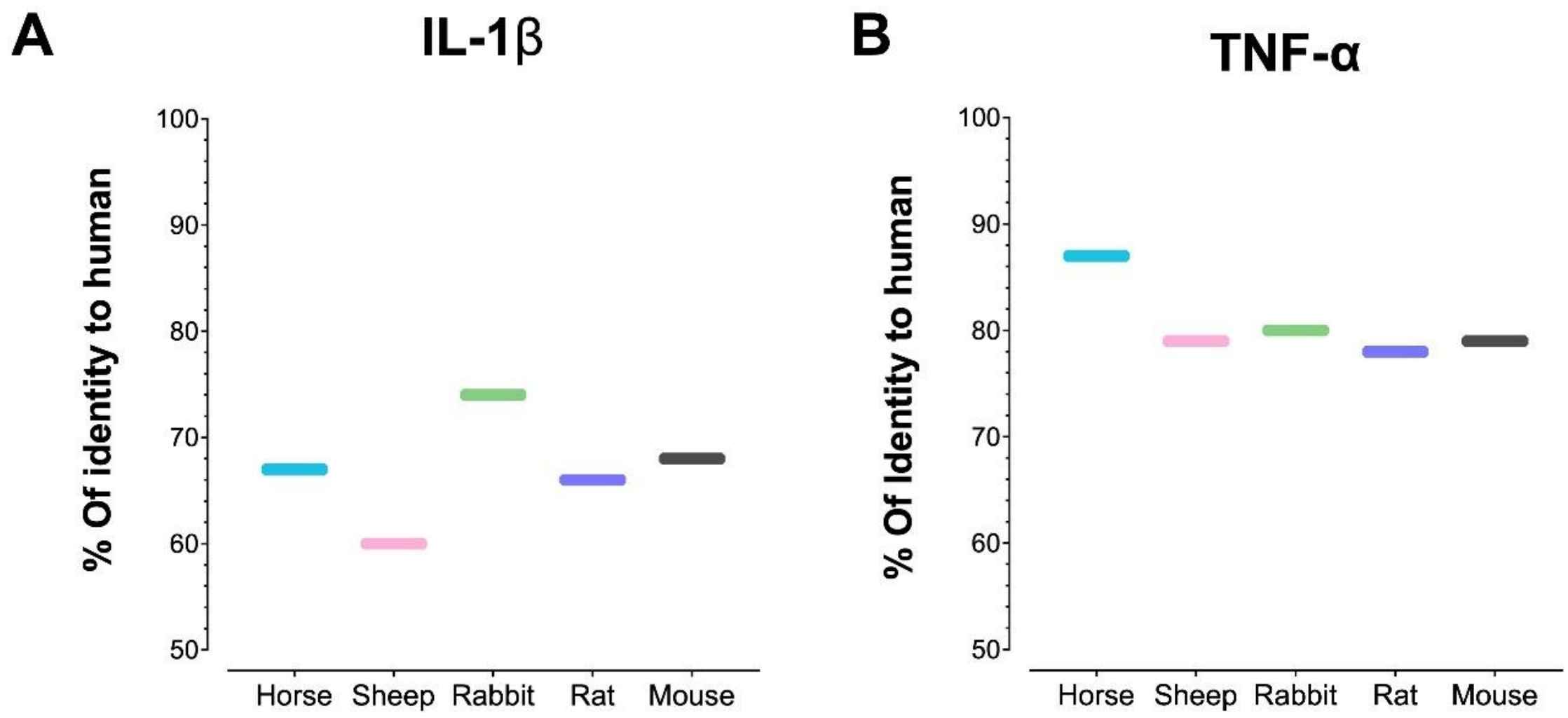

Figure S2. Percentage of identity. Related to methods and discussion.
Graphs show the percentage identity of IL-1β and TNFα sequences from horse, sheep, rabbit, rat, and mouse when BLASTed against the human sequences.

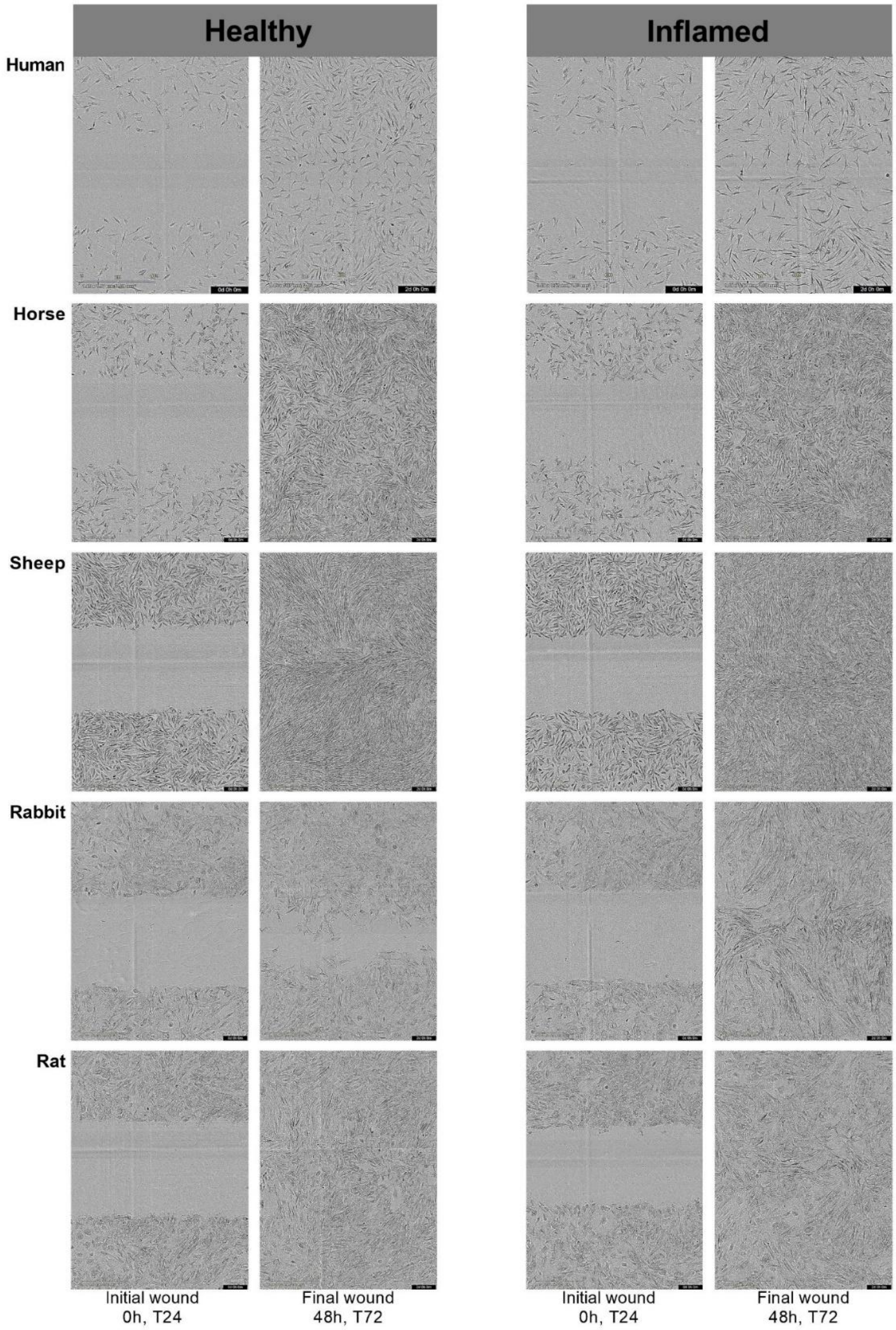

Figure S3. Scratch wound assay comparing healthy and inflamed conditions at the initial and final time points. Related to Figure 3. Chondrocytes from all species were seeded at an identical density of 5,000 cells per well in IncuCyte® ImageLock 96-well plates (Sartorius, Germany). At T24, following the application of the inflammatory treatment, uniform scratch wounds were created using the IncuCyte® WoundMaker (Sartorius, Germany). Initial wound images were acquired 30 minutes after scratching (0 h) to capture the baseline wound area. Final wound images were collected after 48 hours of observation (48 h post-scratch; corresponding to T72 overall). Representative images illustrate wound closure under healthy and inflamed conditions across the five species tested. Cell density in equine wells appears lower than in rabbit wells despite faster equine proliferation (Figure 3) because confluence at any absolute timepoint reflects the product of cell number and per-cell area; as equine chondrocytes are significantly smaller than rabbit chondrocytes (Figure 1), their small per-cell area contribution is not overcome by their proliferative advantage within the 48-hour observation window of the scratch assay. (Representative images from 1 of 3 donors)

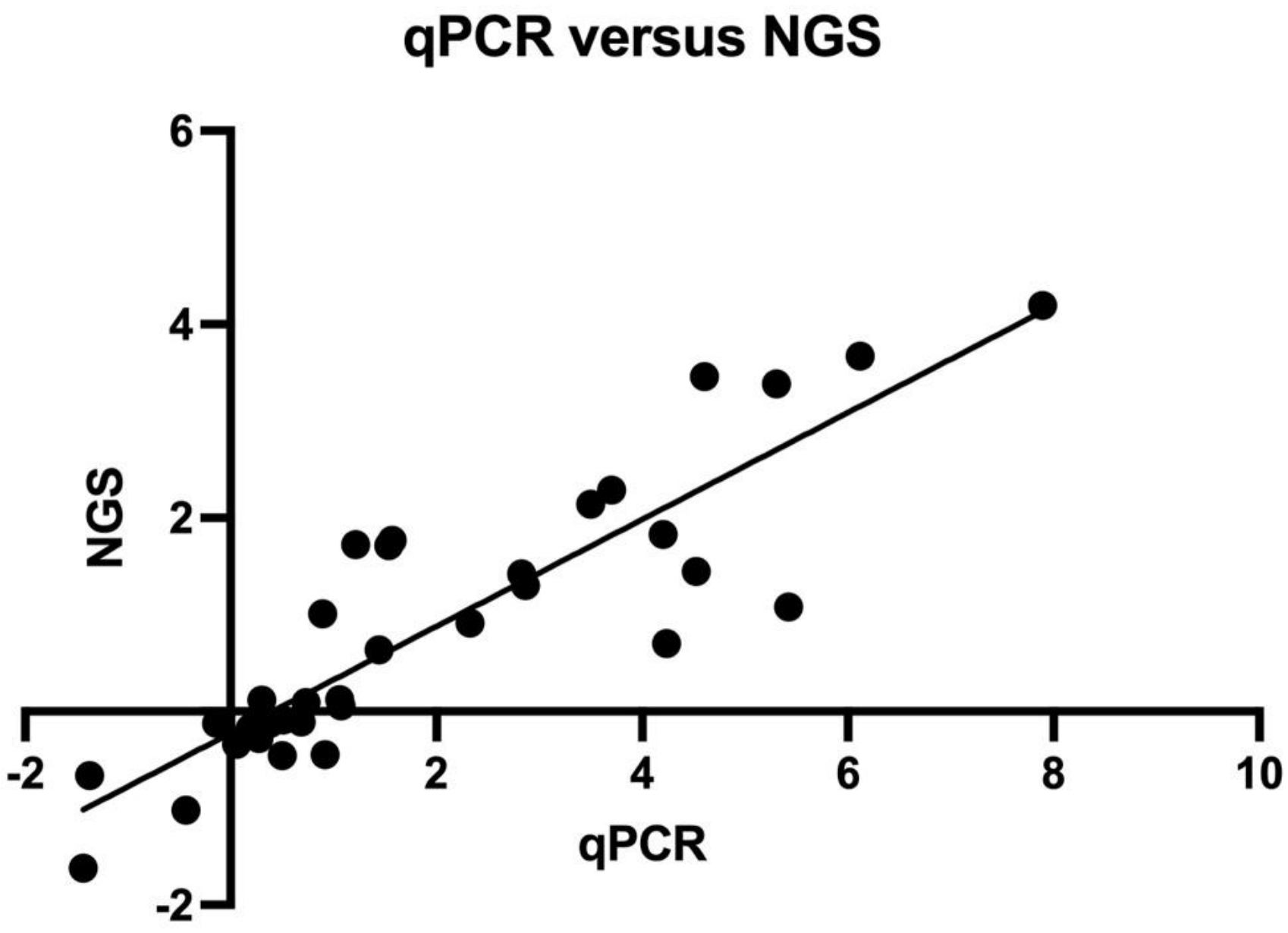

Figure S4. NGS versus qPCR validation scatter plot (related to results and Table S8).

Scatter plot showing the relationship between log_2(fold-change) RNA-seq and qPCR measurements for eight genes (*IL6, MMP1, MMP13, SOD2, TXNRD1, ACAN, COL2A1, PRG4*) across five species (human, horse, sheep, rabbit, rat). A strong positive cross-platform association was observed (Spearman's $\rho = 0.871$, $p < 0.0001$), indicating consistent direction and magnitude of differential expression between methods.

Supplementary Tables

Table S1. Comparative Inflammatory Gene Expression Across Concentrations. Related to Figure 2.

This table summarizes the expression levels of key inflammatory genes across all studied species following stimulation with four different concentrations of inflammatory cytokines. Data are presented as mean values with corresponding statistical significance levels (*p < 0.05, **p < 0.01, ***p < 0.001, ****p < 0.0001), calculated using one-way ANOVA relative to the baseline (healthy control). The results illustrate both species-specific responses and dose-dependent modulation of inflammatory gene expression.

	Human		Horse		Sheep		Rabbit		Rat	
	Mean	SEM	Mean	SEM	Mean	SEM	Mean	SEM	Mean	SEM
IL6										
1ng/mL	3.600**	0.717	2.821	1.069	0.447	0.069	0.927	0.248	0.138	0.158
2.5ng/mL	4.639***	0.658	3.554	0.960	1.188***	0.080	2.055**	0.307	0.679	0.124
5ng/mL	5.382***	0.677	5.606**	1.371	1.858***	0.059	3.030***	0.507	2.211*	0.673
10 ng/mL	5.455***	0.644	7.106**	1.092	2.130****	0.257	3.914***	0.339	2.815*	0.626
MMP1										
1ng/mL	3.851****	0.326	1.679	0.512	3.290***	0.797	2.295	0.986	4.108	1.228
2.5ng/mL	4.740****	0.271	2.804	0.907	3.215***	0.026	3.697**	0.749	8.949**	3.061
5ng/mL	5.409****	0.229	4.803*	1.354	3.708***	0.058	3.971**	0.625	11.507**	0.688
10 ng/mL	5.477****	0.169	5.595**	0.884	3.918****	0.175	4.501**	0.040	13.207***	1.092
MMP13										
1ng/mL	3.666	1.684	2.127	0.602	1.773*	0.305	-0.190	0.273	3.069	1.299
2.5ng/mL	5.350*	1.040	4.261*	0.318	2.480**	0.014	-0.308	0.047	4.232	1.743
5ng/mL	5.988*	1.197	5.902**	1.525	3.261***	0.404	-0.419	0.061	6.056*	1.642
10 ng/mL	6.026*	1.217	4.496*	0.948	3.859***	0.525	-0.452	0.144	6.602*	1.611

Table S7. Shared canonical pathways between human and animal species. Related to Figure 4.

The table summarizes the nine canonical pathways identified as shared between human and the animal species studied. For each pathway, the corresponding z-scores are reported, highlighting conserved molecular mechanisms across species.

**Shared pathways from Ingenuity Pathway Analysis**	**Z-score**			
**Canonical Pathways**	**Human**	**Horse**	**Sheep**	**Rat**
Class A/1 (Rhodopsin-like receptors)	3.771	2		2
Deubiquitination	3	2	2.236	
Acute Phase Response Signaling	2	2		2.449
G alpha (i) signalling events	3.411			2
Neutrophil Extracellular Trap Signaling Pathway	3.266			2
Sheddase Signaling Pathway	2.887			2.449
Role of Macrophages, Fibroblasts and Endothelial Cells in Rheumatoid Arthritis	2.611	2.236		
NRF2-mediated Oxidative Stress Response	2.121	2		
Extracellular matrix organization	-2.236	-2		

Table S8. Comparison of $\log_2$(fold-change) values obtained by RNA-seq and qPCR for eight genes (*IL6, MMP1, MMP13, SOD2, TXNRD1, ACAN, COL2A1, PRG4*) across five species (human, horse, sheep, rabbit, rat), demonstrating consistent directionality of gene expression changes (related to Results).

Gene	Species	qPCR			NGS		
IL6	Human	8.2353	5.6918	4.4258	3.8900	2.8755	4.2541
	Horse	3.8239	7.7999	4.6423	1.5874	1.1476	0.4989
	Sheep	3.4206	3.6784	5.6145	0.6283	0.9506	0.5176
	Rabbit	0.5294	0.2593	-0.6268	0.0625	-0.8748	-0.2080
	Rat	2.2017	2.3828	3.8987	1.7718	1.8909	0.6012
MMP1	Human	5.3754	2.9878	2.1316	1.9911	1.9534	2.4837
	Horse	5.2185	1.5964	7.0066	3.8803	2.0876	4.4191
	Sheep	5.1468	5.4105	5.3608	4.1513	2.9145	3.0874
	Rabbit	0.6816	0.3033	0.5142	0.0384	-1.2996	-0.1097
	Rat	1.6681	4.5179	-3.0176	0.3718	0.0000	0.0000
MMP13	Human	6.6999	3.3647	3.5137	2.0396	0.0165	2.2763
	Horse	2.2489	5.5367	3.3242	2.4017	1.5665	2.8882
	Sheep	7.6456	8.3454	7.6857	4.9470	3.2741	4.3654
	Rabbit	0.8822	1.4892	-0.1826	-0.4605	0.9040	-0.1633
	Rat	0.9719	1.8751	-0.1666	0.6291	2.3715	0.0199
SOD2	Human	2.8941	1.8558	-1.1129	1.8794	1.1532	2.1362
	Horse	1.4261	0.7339	2.5484	1.3289	2.0658	1.9078
	Sheep	3.8303	4.5394	4.2392	1.9216	1.5930	1.9680
	Rabbit	0.7858	1.7305	0.2780	0.0000	0.0000	0.0000
	Rat	1.0018	2.4160	1.1840	1.9276	2.6110	0.5948
TXNRD1	Human	2.2905	1.8239	0.2061	0.2914	1.3172	0.3057
	Horse	0.8983	3.1207	2.9578	0.9469	0.9187	0.8775
	Sheep	0.3241	0.5390	1.1932	0.0082	-0.1258	-0.2107
	Rabbit	1.0784	0.8936	-0.4381	-0.1553	0.0594	-0.1737
	Rat	0.6652	-0.3471	0.5818	-0.0574	0.2039	0.1977
ACAN	Human	1.0244	0.0899	-0.3005	0.4695	-1.0939	-0.2145
	Horse	-0.8786	2.4812	-2.9073	-0.8025	-0.7349	-1.5254
	Sheep	-0.1440	0.3180	2.5700	-0.5203	-0.3159	-0.5098
	Rabbit	0.7104	2.5914	-0.0857	0.0000	0.0000	0.1663
	Rat	0.0080	-0.0650	2.0270	0.0000	0.0000	0.0000
COL2A	Human	0.0177	0.2661	-0.6955	-0.3690	0.3660	-0.3640
	Horse	-0.6163	0.2819	-3.7803	0.0000	0.0000	-1.9929
	Sheep	0.6020	-0.1400	0.0970	-0.2561	0.0000	-0.2708
	Rabbit	0.1053	2.6734	0.2020	0.0000	0.0000	0.0000
	Rat	-0.1140	-0.7310	0.1500	0.0000	0.0000	0.0000
PRG4	Human	-1.8152	-0.8886	-1.5969	-1.6602	-1.3558	-1.8370
	Horse	-0.7417	-2.6557	0.4315	0.0000	0.0000	0.0000
	Sheep	-0.0140	0.2920	0.8660	0.0543	-0.6472	0.2183

	Rabbit	0.5372	3.2088	0.8812	0.0000	0.0000	0.0000
	Rat	3.2000	3.7690	1.6210	0.9667	1.6455	1.2863

Table S10. List of primers pair. Related to Key resources table.

Forward and reverse primer sequences used for RT-qPCR analysis of *IL6, MMP1, MMP13, SOD2*, *TXNRD1, COL2A1, ACAN*, and *PRG4* across all species included in this study.

	**Human**	
	**Forward Primer**	**Reverse Primer**
IL6	CTCAGCCCTGAGAAAGGAGA	AGGTTGTTTTCTGCCAGTGC
MMP1	GCAGCTTCAGTGACAAAC	TTCCTCCGGCAAAGACTCAT
MMP13	TCAGGAAACCAGGTCTGGAG	TGACGCGAACAATACGGTTA
SOD2	GGCTCCGGTTTTGGGGTATC	TTGATGTGAGGTTCCAGGGC
TXNRD1	ATGGCAAGAAGGTGATGGTCC	TCACACATGTTCCTCCGAGAC
COL2A1	TCGTGGCAGAGATGGAGAAC	CCATCTGGGCAGCAAAGTTT
ACAN	ACTGGCGAGCACTGTAACAT	TAGCTGGGAAGGCATAAGCA
PRG4	GCTGCTTTGAGTCCTTCGAG	AAGATGGTGGTGATGTGGGA
GAPDH	ATCCCATCACCATCTTCCAG	TGGACTCCACGACGTACTCA
RPLP0	GGCACCATTGAAATCCTGAGTG	GTAGATGCTGCCATTGTCGAAC
	**Horse**	
	**Forward Primer**	**Reverse Primer**
IL6	ATGGCAGAAAAAGACGGATG	GGGTCAGGGGTGGTTACTTC
MMP1	GCTGTTAAGGGGCAGGATGT	TCACAGTGCTAGGAAAGCCG
MMP13	TGGTCCAGGAGATGAAGACC	GATGGCATCAAGGGATAAGG
SOD2	TCTGGAGCCCTACATCAACG	GGTGACGTTCAGGTTGTTCAC
TXNRD1	GGATCTTCCCGAGTCCTATGA	CAAGAGGGGTTGGAGTGACAA
COL2A1	GGTAACCCTGGAACTGATGGA	AGGACCAGTTGCACCTTGAG
ACAN	GCTCCAATGACTCTGGCATC	GCCTTTCACCACGACCTCTA
PRG4	CCTGTAAAGGTCGCTGCTTC	CACTTGCCGTACTTCTTGCA
GAPDH	GTTTGTGATGGGCGTGAAC	GATGCCAAAGTGGTCATGG
RPLP0	GCTTCATTGTGGGAGCAGAC	TTTTCCAGATGCCCTCGGAT
	**Sheep**	
	**Forward Primer**	**Reverse Primer**
IL6	GTCATGGAGTTGCAGAGCAG	ACCCACTCGTTTGAGGACTG
MMP1	CCCACAAACCCCAGAAGTGT	TGCGTAGAAGGGATTTGTGC
MMP13	TCCTCTTCTTCAGCTGGACG	TCCTCGGACAAGTCTTCAGA
SOD2	ACATCAACGCGCAGATCATG	GCTGTGATTGATATGGCCCC
TXNRD1	TCTTGGAGGAACGTGTGTGA	AGAGTCTCGTAGGGCTTGTC
COL2A1	TGGTGCCAAAGGTGAACAAG	AGGACCAGTCACACCAGTAG
ACAN	CCTTGTCTGAATGGAGCCAC	TCTCACACATGTCCCCTTGG
PRG4	TCACTGCCCAACGTCAGAAA	TGGTCACCATTGAACACTTGG
GAPDH	AGATGGTGAAGGTCGGAGTG	GAAGGTCAATGAAGGGGTCA
RPLP0	GAACACGATGATGCGCAAGG	ACATTTCCCCGGATGTGAGG
	**Rabbit**	
	**Forward Primer**	**Reverse Primer**
IL6	AGGAGCTGAGGAAAGAGATGT	TCCGTCTTCTTCTATCAGCCTT
MMP1	TTCCCAGCAGCTTCAGAAAC	GCCCAAAGAATTCCTGCATTTG

MMP13	ACCATCCTCTGAATCCTGCG	AAGACTGCATTTCTCGGAGC
SOD2	AGAGCCCAAAGGGGAGTTAC	TCCTTATTGAAGCCGAGCCA
TXNRD1	GACCTCATCATCATTGGCGG	CAAGAGGAGTTGGGGTGACA
COL2A1	TGAAGATGGTCGCCCTGGA	AGTCCCTTCTCACCAGCTTT
ACAN	CTCCAATGACTCCGGGATCT	GAGATGGCCCGATAGTGGAA
PRG4	TACTCAGTGTATGGGCAGGC	GTGTCTGGAAAGGTCCAACG
GAPDH	AGGTCGGAGTGAACGGATT	CGACAACATCCACTTTGCCA
RPLP0	TGGGCAAGAACACCATGATG	TTTCCCCGGATATGAGGCAG
	**Rat**	
	**Forward Primer**	**Reverse Primer**
Il6	TGTATGAACAGCGATGATG	AGAAGACCAGAGCAGATT
Mmp1	GCC CAT ACA GTT TGA ATA- CAG TATCTG	CCA GTT TAA TAA ACA CCA TCT CTT GA
Mmp13	CCCTCGAACACTCAAATGGT	AAGGCCTTCTCCACTTCAGA
Sod2	TCACCGAGGAGAAGTACCAC	CTTAGGGCTCAGGTTTGTCC
Txnrd1	CGGGAGAAGAAGGTTGTCTATG	GCAGTACTCTTTGTCTCCAGG
Col2a1	AAGATGGTCCCAAAGGTGCT	GGTTCGCCTTTCTCTCCAGG
Acan	ACCTTCGCTCCAATGACTCT	TCGGTCAAAGTCCAGTGTGT
Prg4	ACCTTTCCAGACACACACCT	ATGTTGGGCAGTGTTATCGC
Gapdh	GGCACAGTCAAGGCTGAGAATG	ATGGTGGTGAAGACGCCAGTA
Rplp0	CAGCATCTACAGCCCAGAGG	CAAAGCCAGGACCCTTTTGT
